# Evaluation of Combined Artificial Intelligence and Radiologist Assessment to Interpret Screening Mammograms

**DOI:** 10.1001/jamanetworkopen.2020.0265

**Published:** 2020-03-02

**Authors:** Thomas Schaffter, Diana S. M. Buist, Christoph I. Lee, Yaroslav Nikulin, Dezső Ribli, Yuanfang Guan, William Lotter, Zequn Jie, Hao Du, Sijia Wang, Jiashi Feng, Mengling Feng, Hyo-Eun Kim, Francisco Albiol, Alberto Albiol, Stephen Morrell, Zbigniew Wojna, Mehmet Eren Ahsen, Umar Asif, Antonio Jimeno Yepes, Shivanthan Yohanandan, Simona Rabinovici-Cohen, Darvin Yi, Bruce Hoff, Thomas Yu, Elias Chaibub Neto, Daniel L. Rubin, Peter Lindholm, Laurie R. Margolies, Russell Bailey McBride, Joseph H. Rothstein, Weiva Sieh, Rami Ben-Ari, Stefan Harrer, Andrew Trister, Stephen Friend, Thea Norman, Berkman Sahiner, Fredrik Strand, Justin Guinney, Gustavo Stolovitzky, and the DM DREAM Consortium, Lester Mackey, Joyce Cahoon, Li Shen, Jae Ho Sohn, Hari Trivedi, Yiqiu Shen, Ljubomir Buturovic, Jose Costa Pereira, Jaime S. Cardoso, Eduardo Castro, Karl Trygve Kalleberg, Obioma Pelka, Imane Nedjar, Krzysztof J. Geras, Felix Nensa, Ethan Goan, Sven Koitka, Luis Caballero, David D. Cox, Pavitra Krishnaswamy, Gaurav Pandey, Christoph M. Friedrich, Dimitri Perrin, Clinton Fookes, Bibo Shi, Gerard Cardoso Negrie, Michael Kawczynski, Kyunghyun Cho, Can Son Khoo, Joseph Y. Lo, A. Gregory Sorensen, Hwejin Jung

**Affiliations:** 1 Computational Oncology, Sage Bionetworks, Seattle, Washington; 2 Kaiser Permanente Washington Health Research Institute, Seattle, Washington; 3 University of Washington School of Medicine, Seattle; 4 Therapixel, Paris, France; 5 Department of Physics of Complex Systems, ELTE Eötvös Loránd University, Budapest, Hungary; 6 Department of Computational Medicine and Bioinformatics, Michigan Medicine, University of Michigan, Ann Arbor; 7 DeepHealth Inc, Cambridge, Massachusetts; 8 Tencent AI Lab, Shenzhen, China; 9 National University of Singapore, Singapore; 10 Integrated Health Information Systems Pte Ltd, Singapore; 11 Department of Electrical and Computer Engineering, National University of Singapore, Singapore; 12 National University Health System, Singapore; 13 Lunit Inc, Seoul, Korea; 14 Instituto de Física Corpuscular (IFIC), CSIC–Universitat de València, Valencia, Spain; 15 Universitat Politecnica de Valencia, Valencia, Valenciana, Spain; 16 Centre for Medical Image Computing, University College London, Bloomsbury, London, United Kingdom; 17 Tensorflight Inc, Mountain View, California; 18 University of Illinois at Urbana-Champaign, Urbana; 19 IBM Research Australia, Melbourne, Australia; 20 IBM Research Haifa, Haifa University Campus, Mount Carmel, Haifa, Israel; 21 Stanford University, Stanford, California; 22 Department of Biomedical Data Science, Radiology, and Medicine (Biomedical Informatics), Stanford University, Stanford, California; 23 Department of Physiology and Pharmacology, Karolinska Institutet, Stockholm, Sweden; 24 Department of Diagnostic, Molecular and Interventional Radiology, Icahn School of Medicine at Mount Sinai, New York, New York; 25 Department of Pathology, Molecular and Cell-Based Medicine, Icahn School of Medicine at Mount Sinai, New York, New York; 26 Department of Genetics and Genomic Sciences, Icahn School of Medicine at Mount Sinai, New York, New York; 27 Department of Population Health Science and Policy, Department of Genetics and Genomic Sciences, Icahn School of Medicine at Mount Sinai, New York, New York; 28 Fred Hutchinson Cancer Research Center, Seattle, Washington; 29 Bill and Melinda Gates Foundation, Seattle, Washington; 30 Center for Devices and Radiological Health, Food and Drug Administration, Silver Spring, Maryland; 31 Department of Oncology-Pathology, Karolinska Institutet, Stockholm, Sweden; 32 Breast Radiology, Karolinska University Hospital, Stockholm, Sweden; 33 IBM Research, Translational Systems Biology and Nanobiotechnology, Thomas J. Watson Research Center, Yorktown Heights, New York; 34 Microsoft New England Research and Development Center, Cambridge, Massachusetts; 35 North Carolina State University, Raleigh; 36 Icahn School of Medicine at Mount Sinai, New York, New York; 37 Department of Radiology and Biomedical Imaging, University of California, San Francisco, San Francisco; 38 Emory University, Atlanta, Georgia; 39 New York University, New York; 40 Clinical Persona, East Palo Alto, California; 41 Institute for Systems and Computer Engineering, Technology and Science, Porto, Portugal; 42 KolibriFX, Oslo, Norway; 43 Department of Computer Science, University of Applied Sciences and Arts, Dortmund, Germany; 44 Department of Diagnostic and Interventional Radiology and Neuroradiology, University Hospital Essen, Essen, Germany; 45 Biomedical Engineering Laboratory Tlemcen University, Tlemcen, Algeria; 46 Department of Radiology, NYU School of Medicine, New York, New York; 47 Queensland University of Technology, Brisbane, Australia; 48 MIT-IBM Watson AI Lab, IBM Research, Cambridge, Massachusetts; 49 Institute for Infocomm Research, A*STAR, Singapore; 50 Icahn Institute for Data Science and Genomic Technology, New York, New York; 51 Carl E. Ravin Advanced Imaging Laboratories, Department of Radiology, Duke University School of Medicine, Durham, North Carolina; 52 Satalia, London, United Kingdom; 53 Bakar Computational Health Sciences Institute, University of California, San Francisco, San Francisco; 54 University College London, London, United Kingdom; 55 Department of Radiology, Duke University School of Medicine, Durham, North Carolina; 56 Korea University, Seoul, Korea

## Abstract

**Importance:**

Mammography screening currently relies on subjective human interpretation. Artificial intelligence (AI) advances could be used to increase mammography screening accuracy by reducing missed cancers and false positives.

**Objective:**

To evaluate whether AI can overcome human mammography interpretation limitations with a rigorous, unbiased evaluation of machine learning algorithms.

**Design, Setting, and Participants:**

In this diagnostic accuracy study conducted between September 2016 and November 2017, an international, crowdsourced challenge was hosted to foster AI algorithm development focused on interpreting screening mammography. More than 1100 participants comprising 126 teams from 44 countries participated. Analysis began November 18, 2016.

**Main Outcomes and Measurements:**

Algorithms used images alone (challenge 1) or combined images, previous examinations (if available), and clinical and demographic risk factor data (challenge 2) and output a score that translated to cancer yes/no within 12 months. Algorithm accuracy for breast cancer detection was evaluated using area under the curve and algorithm specificity compared with radiologists’ specificity with radiologists’ sensitivity set at 85.9% (United States) and 83.9% (Sweden). An ensemble method aggregating top-performing AI algorithms and radiologists’ recall assessment was developed and evaluated.

**Results:**

Overall, 144 231 screening mammograms from 85 580 US women (952 cancer positive ≤12 months from screening) were used for algorithm training and validation. A second independent validation cohort included 166 578 examinations from 68 008 Swedish women (780 cancer positive). The top-performing algorithm achieved an area under the curve of 0.858 (United States) and 0.903 (Sweden) and 66.2% (United States) and 81.2% (Sweden) specificity at the radiologists’ sensitivity, lower than community-practice radiologists’ specificity of 90.5% (United States) and 98.5% (Sweden). Combining top-performing algorithms and US radiologist assessments resulted in a higher area under the curve of 0.942 and achieved a significantly improved specificity (92.0%) at the same sensitivity.

**Conclusions and Relevance:**

While no single AI algorithm outperformed radiologists, an ensemble of AI algorithms combined with radiologist assessment in a single-reader screening environment improved overall accuracy. This study underscores the potential of using machine learning methods for enhancing mammography screening interpretation.

## Key Points

**Question:**

How do deep learning algorithms perform compared with radiologists in screening mammography interpretation?

**Findings:**

In this diagnostic accuracy study using 144 231 screening mammograms from 85 580 women from the United States and 166 578 screening mammograms from 68 008 women from Sweden, no single artificial intelligence algorithm outperformed US community radiologist benchmarks; including clinical data and prior mammograms did not improve artificial intelligence performance. However, combining best-performing artificial intelligence algorithms with single-radiologist assessment demonstrated increased specificity.

**Meaning:**

Integrating artificial intelligence to mammography interpretation in single-radiologist settings could yield significant performance improvements, with the potential to reduce health care system expenditures and address resource scarcity experienced in population-based screening programs.

## Teaser

This diagnostic accuracy study evaluates whether artificial intelligence can overcome human mammography interpretation limits with a rigorous, unbiased evaluation of machine learning algorithms.

## Introduction

Mammography screening is one of the most widely deployed tools for early breast cancer detection and has been shown to decrease mortality in multiple randomized clinical trials.^1^ However, screening mammography is imperfect with 1 in 8 cancers missed at time of interpretation in US community practice.^2^ Roughly 9% to 10% of the 40 million US women who undergo routine breast screening each year are recalled for additional diagnostic imaging; only 4% to 5% of women recalled are ultimately diagnosed as having breast cancer.^2^ These false positives lead to preventable harms, included patient anxiety, benign biopsies, and unnecessary intervention or treatment.^3^ High false-positive rates incur significant resources and contribute to the annual $10 billion mammography screening costs in the United States.^4^

Currently, mammograms are interpreted by radiologists and rely on human visual perception to identify relevant traits,^5^ leaving its benefit dependent on subjective human interpretation to maximally extract all diagnostic information from the acquired images.^6^ In 1998, computer-assisted detection software was developed for mammography with the hopes of improving radiologist performance; however, computer-assisted detection has not improved interpretive accuracy.^7,8^ Recent deep learning advances, and the ever increasing large computational power and digital mammography (DM) data availability, renewed the interest in evaluating whether more sophisticated models based on quantitative imaging features can match or even outperform human interpretation alone.^9,10,11,12,13,14,15,16,17^ Such efforts could aid in improving specificity and overall performance in single-radiologist settings. In double-radiologist interpretive settings such as in Europe, highly accurate algorithms could alleviate the person power needed for double-radiologist interpretation and consensus.

Throughout the last decade, crowdsourced competitions or challenges have been popularized as highly effective mechanisms for engaging the international scientific community to solve complex scientific problems.^18,19^ The Dialogue on Reverse Engineering Assessment and Methods (DREAM) initiative has run dozens of biomedical challenges, establishing robust and objective computational benchmarks in multiple disease areas and across multiple data modalities.^19^ This report describes the DM DREAM challenge, which was designed to develop and validate breast cancer detection algorithms to determine whether machine learning methods applied to mammography data can improve screening accuracy.^20,21^

## Methods

The study followed the Standards for Reporting of Diagnostic Accuracy (STARD) reporting guideline.^22^ We conducted an international crowdsourced challenge to assess whether artificial intelligence (AI) algorithms could meet or beat radiologists’ interpretive screening mammography performance.^21^ The challenge asked participants to develop algorithms inputting screening mammography data and outputting a score representing the likelihood that a woman will be diagnosed with breast cancer within the next 12 months (eAppendix 2 in the Supplement). Digital mammogram images included different views (eTable 2 in the Supplement) from the most recent screening examination. Subchallenge 2 provided access to images for the current and, when available, previous screening examinations, as well as clinical and demographic risk factor information typically available to interpreting radiologists (eAppendix 3 and eTable 1 in the Supplement).

The DM DREAM challenge was hosted between November 2016 and November 2017. There were 4 phases (eAppendix 1 and eFigure 1 in the Supplement): open phase (September 2016-November 2016), leaderboard phase (November 2016-March 2017), and validation phase (March 2017-May 2017), which together constitute the competitive phase and the community phase (July 2017-November 2017). A first data set was provided by Kaiser Permanente Washington (KPW) (eAppendix 5 in the Supplement), which was used during the competitive and community phases. A second data set was provided by the Karolinska Institute (KI) in Sweden (eAppendix 5 in the Supplement), which was only used for trained algorithm validation. To protect data privacy, both data sets were securely protected behind a firewall and were not directly accessible to challenge participants, who had to submit their algorithms for automated training and testing behind the firewall (eAppendix 7 in the Supplement).

**Figure 1.**
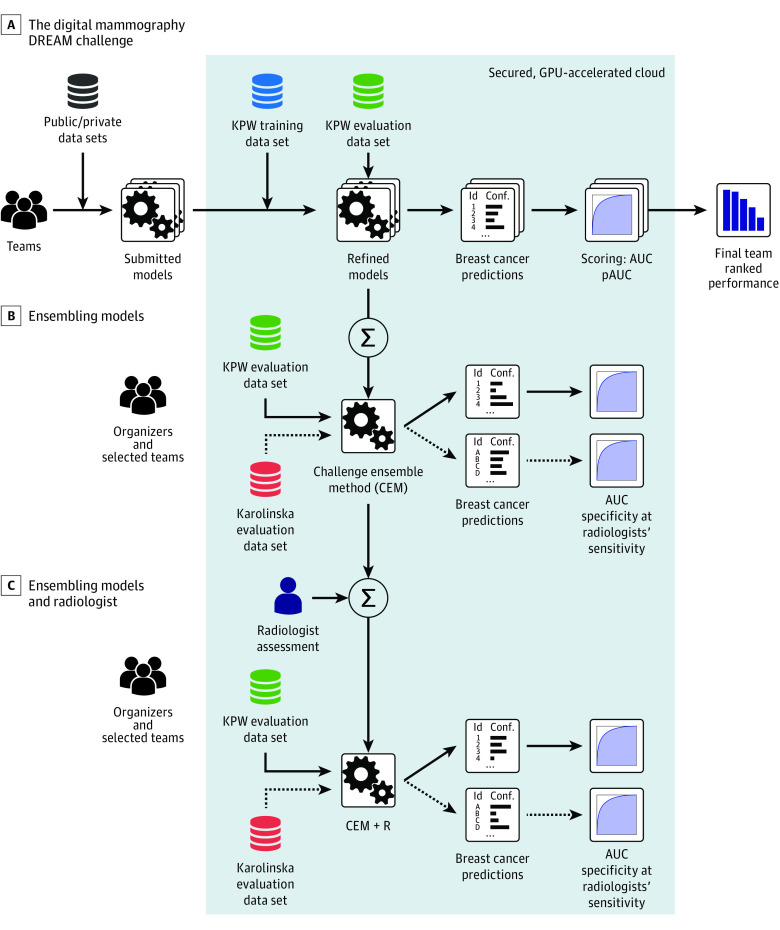
Training and Evaluation of Algorithms During the Digital Mammography DREAM Challenge Training and evaluation Kaiser Permamente Washington (KPW) and Karolinskia Institute data were not directly available to challenge participants; they were stored behind a firewall in a secure cloud (gray box). To access the data, participants submitted models to be run behind the firewall, in the graphics processing unit (GPU)–accelerated cloud (IBM). A, Training and evaluation of models submitted by teams during the Digital Mammography Dialogue on Reverse Engineering Assessment and Methods (DREAM) Challenge. B, A subset of the 8 best models in the evaluation KPW dataset were combined into the Challenge Ensemble Method (CEM), trained using the KPW training set and evaluated in the KPW and Karolinska Institute evaluation datasets. C, We developed a final ensemble method incorporating radiologists' interpretation in a method called CEM+radiologist (CEM+R). AUC indicates area under the curve; pAUC, partial area under the curve.

In the competitive phase, the KPW data set was randomly split into 3 data sets matched on age, body mass index, and race/ethnicity (eAppendix 3 in the Supplement): leaderboard phase training (50%), leaderboard phase evaluation (20%) (eTable 3 in the Supplement), and final evaluation data set (30%) (eTable 4 in the Supplement). The leaderboard phase allowed competitors to train algorithms using the KPW training data or external (public or private) data and submit their trained algorithms for evaluation. To minimize overfitting, a maximum of 9 submissions per team were scored in the leaderboard data set with publicly posted results.^20^ During the validation phase, participants were scored using the KPW final evaluation data set (Figure 1A) using area under the curve (AUC) and partial AUC as evaluation metrics (eAppendix 6 in the Supplement) assessed at the examination level, on which the final ranking of team performances was determined.

The 8 top-performing competitive phase teams were invited to collaborate during the community phase to further refine their algorithms (Figure 1B). The output of this phase was an ensemble model, consisting of a weighted aggregation of algorithm predictions into a new algorithm called the *challenge ensemble method* (CEM). The CEM model was further integrated with the radiologists’ assessment into another ensemble model called the *CEM+R model*. The CEM and CEM+R models were trained using the training and leaderboard data of the competitive phase, with performance assessed using the KPW and KI final evaluation data sets (Figure 1C and eTable 5 in the Supplement).

### Data Sources and Characteristics

Kaiser Permamente Washington provided the primary data set for the challenge, with images linked to curated data as part of the Breast Cancer Surveillance Consortium.^21^ These data include prospectively collected patient-reported breast cancer risk factor information linked to breast imaging data, radiologist interpretations and recommendations, and benign and malignant breast tumor biopsy results. An additional independent validation data set was provided by the KI and comprised women screened in the Stockholm region of Sweden between April 2008 to December 2012 with comparable data provided by KPW. Both data sets were deidentified full-field DMs. This collaboration received institutional review board approval at Sage Bionetworks with a waiver of consent and Health Insurance Portability and Accountability Act.

Each screening examination was labeled as cancer negative or cancer positive at the breast level, defined as a tissue biopsy yielding an invasive cancer or ductal carcinoma in situ positive result within 1 year of the screening examination. Images were weakly labeled, meaning the presence or absence of cancer was reported per screening examination but not the actual location of cancer on each image. Breast-level performance was used in the competitive phase, whereas examination-level performance was used in the collaborative phase to make results directly comparable to radiologists’ performance (eAppendix 4, eAppendix 6, and eFigure 2 in the Supplement).

### Training and Evaluating Models

Challenge data providers required the data to be protected from being downloaded, viewed, or otherwise directly accessed by participants. Therefore, we used a model-to-data approach^23^ that required participants to submit containerized algorithms (ie, software that was executed on a participant’s behalf in a secure cloud environment) using the IBM and Amazon cloud platforms. Both clouds were donated especially for this challenge (eAppendix 7 and eFigures 3-5 in the Supplement). The model-to-data system was implemented in Synapse (Sage Bionetworks),^24^ a web-based platform for running biomedical challenges. Participants were asked to structure their models in the form of a lightweight portable machine image called a Docker image.^25^

### Ensemble Method

It has been shown that aggregating the predictions of a set of heterogeneous algorithms can improve performance over the individual algorithms.^26,27^ We developed the CEM and CEM+R ensemble classifiers using a meta-learner stacking method^26^ (Appendix 8 in the Supplement). Given the data for a screening examination, each individual algorithm outputs a confidence level (a number between 0 and 1) indicative of the likelihood estimated by the algorithm that the woman will develop breast cancer within a year of the screening test. The CEM ensemble algorithm takes as inputs the confidence levels of each of the community phase 8 top-performing methods. The CEM+R takes the same inputs as the CEM ensemble plus the radiologist assessment, represented with a 1 if the woman was recalled or a 0 otherwise. This input information is combined to generate the CEM or the CEM+R ensemble prediction. For the meta-learner classifier, we chose an elastic net regularized logistic regression^28^ and the R package caret for construction (R Foundation for Statistical Computing).^29^ We trained the meta-learner (ie, tuned the parameters of the logistic regression) on the KPW training data using 10-fold crossvalidation. Final performance assessment was done by applying exactly once the CEM and CEM+R methods to the evaluation KPW and KI data sets.

### Statistical Analysis

Analysis began November 18, 2016. We used AUC as our primary metric for evaluating and ranking algorithm performance during the competitive phase. To assess the algorithms’ sensitivity and specificity, we computed the radiologists’ sensitivity for the data set under study: 85.9% for KPW and 77.1% (single reader) and 83.9% (consensus reading) for KI, which served as the algorithms’ specificity prediction threshold. Spearman correlation was used to test for rank distribution similarity of AUCs and specificities between the data sets. We used binomial proportion CIs to compare the significance between specificity of radiologists and CEM+R. The DeLong test of significance was used to compare the area under the receiver operating characteristic curve of 2 correlated receiver operating characteristic curves. One-tailed *P *values had a statistical significance threshold of .05. All analyses were completed in R statistical software, version 3.5.1 (R Foundation for Statistical Computing).

## Results

After curation (eAppendix 1 in the Supplement), the KPW data set included 144 231 examinations from 85 580 women, of whom 952 (1.1%) were cancer positive (697 cancers [73.2%] were invasive) (Table). The portion of this data set used to evaluate the methods amounts to 30%, which corresponds to the data of 25 657 women, of whom 283 (1.1%) were cancer positive (202 cancers [71.3%] were invasive), while the remaining examinations were used for training. The KI data set provided 166 578 examinations from 68 008 women, of whom 780 (1.1%) were cancer positive (681 cancers [87.3%] were invasive). Women from the KI data set were younger than those in KPW (mean [SD] age, 53.3 [9.4] years vs 58.4 [9.7] years, respectively). Time between screening examinations was bimodal in the 2 data sets and tended to be longer in the KI set (mean [SD] time for first mode: 18.9 [0.9] months; second mode: 24.9 [1.1] months) vs the KPW set (mean [SD] time for first mode: 12.9 [1.7] months; second mode: 24.2 [2.1] months).

**Table.** Composition of the Data Sets From Kaiser Permanente Washington and Karolinska Institute

Characteristic	No. (%)		
	Kaiser Permanente Washington		Karolinska Institute Evaluation
	Training	Evaluation	
Screening examinations, No.^a^	100 974	43 257	166 578
Women, No.^b^	59 923	25 657	68 008
Women diagnosed with breast cancer within 12 mo of mammogram	669 (1.1)	283 (1.1)	780 (1.1)
Women without a breast cancer diagnosis within 12 mo of mammogram	59 254 (98.9)	25 374 (98.9)	67 228 (98.9)
Invasive breast cancers	495 (74.0)	202 (71.4)	681 (87.3)
Ducal carcinoma in situ	174 (26.0)	81 (28.6)	99 (12.7)
Age, mean (SD), y	58.4 (9.7)	58.4 (9.7)	53.3 (9.4)
BMI, mean (SD)	28.2 (6.9)	28.1 (6.8)	NA
Women with ≥1 prior mammogram	27 165 (45.3)	11 651 (45.4)	50 358 (74.2)
Time since last mammogram, mean (SD), mo			
Mode 1	12.8 (1.7)	12.9 (1.7)	18.9 (0.9)
Mode 2	24.2 (2.1)	24.2 (2.1)	24.9 (1.1)

Abbreviations: BMI, body mass index (calculated as weight in kilograms divided by height in meters squared); NA, not applicable.

^a^ Subchallenge 2 provided access to all screening images for the most recent screening examination and, when available, previous screening examinations.

^b^ Subchallenge 1 provided access only to the digital mammogram images from the most recent screening examination.

### Submitted Algorithms

**Figure 2.**
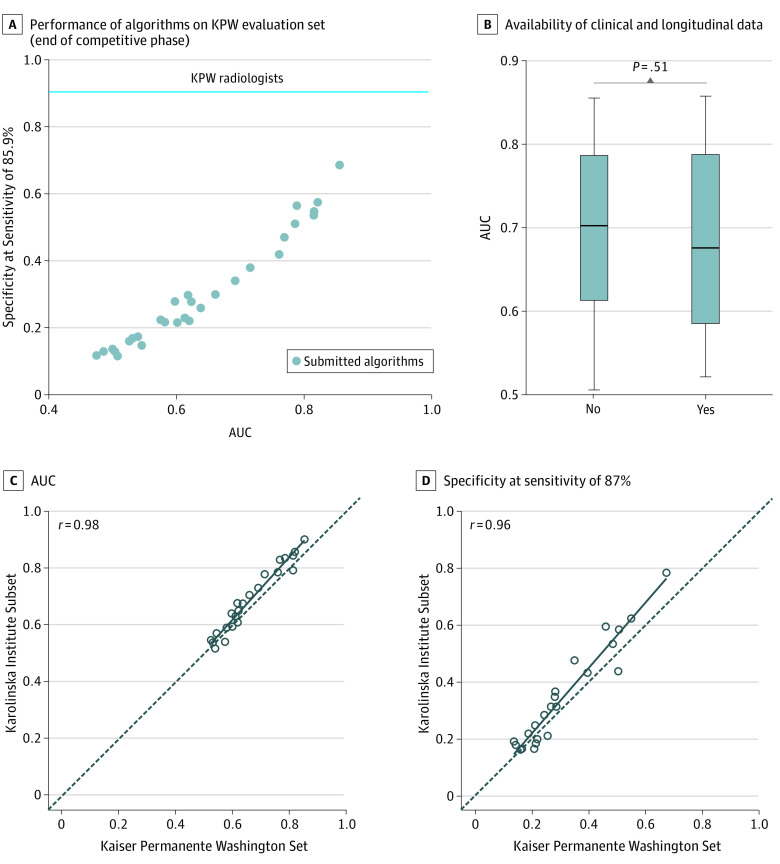
Performance of the Algorithms Submitted at the End of the Competitive Phase Individual algorithm performance submitted at the end of the competitive phase on Kaiser Permamente Washington (KPW) and Karolinska Institute data. A, Area under the curve (AUC) and specificity computed at KPW radiologists' sensitivity of 85.9% of 31 methods submitted to the Digital Mammography Digital Mammography Dialogue on Reverse Engineering Assessment and Methods Challenge and evaluated on KPW evaluation set. B, The performance of methods is not significantly higher when clinical, demographic, and longitudinal data are provided. C-D, The AUC and specificity computed at Breast Cancer Surveillance Consortium’s sensitivity of 86.9% of methods evaluated on the KPW evaluation set generalize to the Karolinska Institute data.

The DM DREAM challenge included more than 1100 individuals participating, comprising 126 teams from 44 countries (eAppendix 9 in the Supplement). Thirty-one teams submitted their methods for final validation in the competitive phase on the KPW evaluation set (Figure 2A). In subchallenge 1, median AUC performance for all teams was 0.611 (interquartile range, 0.54-0.77), with the best-performing method achieving a 0.855 AUC and a specificity of 68.5% at sensitivity of KPW radiologists of 85.9%. The AUC had little improvement when algorithms were able to use clinical, demographic, and longitudinal information (AUC = 0.858 with specificity = 66.3% at sensitivity 85.9%). We observed no improvement across teams in performance measured by AUC (*P* = .51; *t* = 0.024) when comparing their results in subchallenge 2 to subchallenge 1 (Figure 2B).

To assess the generalizability of these methods, we evaluated the top 20 methods on the KI data set. The best-performing method on the KPW data achieved top performance on KI data (AUC = 0.903; specificity = 81.2% at the 83.9% KI radiologists’ sensitivity). Ranking individual methods on the data sets were found to be significantly correlated by AUC (*r* = 0.98; 95% CI, 0.95-1.00; *P* < .001; Figure 2C) and by specificity at Breast Cancer Surveillance Consortium’s average sensitivity^2^ of 86.9% (*r* = 0.96; 95% CI, 0.92-1.00; *P* < .001; Figure 2D).

### Ensemble Models and Radiologists’ Predictions

**Figure 3.**
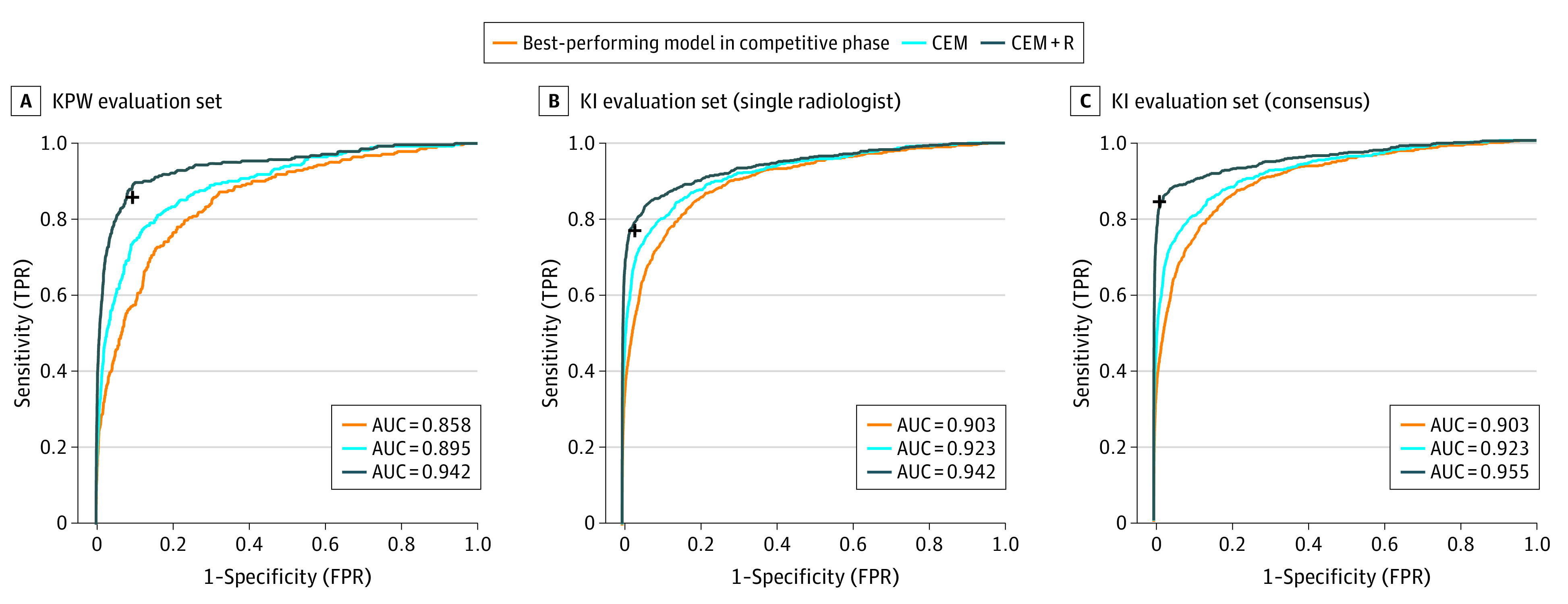
Receiver Operating Characteristic Curves of the Best Individual CEM and CEM+R Methods Receiver operating characteristic curves of the best individual method (orange), challenge ensemble method (CEM) (light blue), and challenge ensemble method + radiologist (CEM+R) method (dark blue) in Kaiser Permamente Washington (KPW) (A) and Karolinska Institute (KI) (B-C) data sets for single radiologist and consensus. The black cross reports the sensitivity and specificity achieved by the radiologist(s) in the corresponding cohort. AUC indicates area under the curve; FPR, false-positive rate; TPR, true-positive rate.

**Figure 4.**
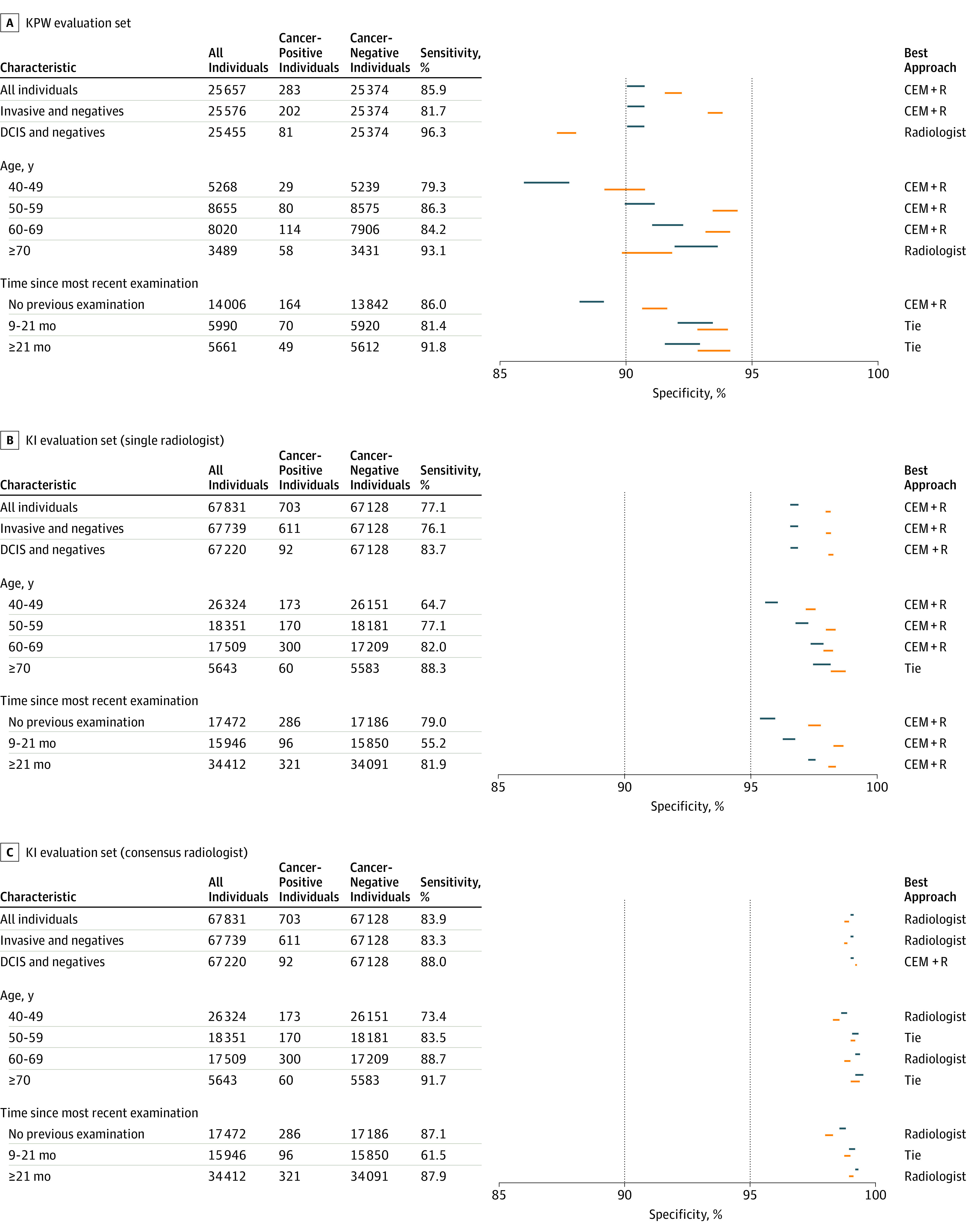
Comparison of the Specificity of Radiologist(s) and CEM+R on Kaiser Permanente Washington (KPW) and Karolinska (KI) Data Comparison of the specificity of radiologist(s) and challenge ensemble method + radiologist (CEM+R) for different clinical/demographic conditions on KPW and KI data. For each condition, we report the CI of the specificity of radiologist (blue) and CEM+R (orange) computed at the sensitivity of radiologists. A best performing approach can be identified when the 2 CIs do not overlap. DCIS indicates ductal carcinoma in situ.

We evaluated whether an ensemble approach could improve overall performance accuracy. Focusing first on the KPW evaluation data, the CEM significantly increased performance (AUC = 0.895; *P* < .001; *z* = 6.7) when compared with the best-performing team (AUC = 0.858) (Figure 3A). To compare the dichotomous screening interpretation (recall/no recall) of the radiologist with continuous AI predictions, we examined the specificity using the fixed sensitivity of the radiologist in each of the cohorts. At the KPW radiologist sensitivity of 85.9%, the specificity of the top model, CEM, and radiologist was 66.3%, 76.1%, and 90.5%, respectively (Figure 4A). Because AI was consistently inferior to the radiologists’ performance, we evaluated whether CEM+R could improve performance. Evaluating the CEM+R on KPW data yielded an AUC of 0.942, with 92% specificity (95% CI, 91.7%-92.3%) (Figure 3A and Figure 4A), higher than the radiologists’ specificity of 90.5% (95% CI, 90.1%-90.9%; *P* < .001).

Accuracy assessments of the CEM and CEM+R models were repeated in patient subpopulations by invasive vs ductal carcinoma in situ, age group, and time since examination. We observed that the CEM+R model consistently resulted in a significantly higher specificity compared with the radiologists’ assessments alone except for women with ductal carcinoma in situ in KPW (Figure 4A), women with at least 1 previous mammogram done 9 months or earlier in KPW (resulting in a tie between radiologists and CEM+R) (Figure 4A) and women in the oldest age groups in both KPW and KI evaluation data sets (Figure 4A and Figure 4B).

Because the KI data set includes data from a countrywide screening program that completes biennial screening with each mammogram undergoing double reading by 2 radiologists, we used the first KI reader interpretation to directly compare with the US data set. Like the KPW analysis, the CEM method achieved a higher AUC (0.923) compared with the top-performing model AUC (0.903) (Figure 3B) on the KI data set. At the first readers’ sensitivity of 77.1%, the specificity of the top model, CEM, and radiologist was 88%, 92.5%, and 96.7%, respectively (Figure 4B). The CEM+R specificity was 98.5% (95% CI, 98.4%-98.6%) (KI AUC: 0.942; Figure 3B and Figure 4B), higher than the radiologist alone specificity of 96.7% (95% CI, 96.6%-96.8%; *P* < .001).

We evaluated whether our ensemble method could be generalized to the double-reading context. Using consensus readings (double reading) from the KI data set, we found sensitivity and specificity of 83.9% and 98.5%, respectively, which outperformed the first readers’ sensitivity and specificity of 77.1% and 96.7%, respectively. The CEM+R algorithm, using the consensus readers’ calls, did not significantly improve the consensus interpretations alone (98.1% vs 98.5% specificity, respectively). This observation persisted in subpopulation analyses, where consensus radiologist interpretations outperformed the CEM+R ensemble across nearly all subpopulations (Figure 4C).

### Top-Performing Algorithmic Methods

The most accurate competitive phase solution was a custom neural network designed for the challenge, initially trained on strongly labeled external data, and subsequently refined using the challenge training data set with 3 teams tied for second (Therapixel model in eAppendix 10 and eFigures 6 and 7 in the Supplement). A second strategy was an adaptation of the Faster R-CNN^30^ object detection framework for mammography,^31^ which was only trained on external data sets with location annotation for lesions (Dezso Ribli’s model in eFigure 8 in the Supplement). Another model used a combination of a higher resolution method to detect calcifications with lower resolution method for masses. A fourth method used a custom neural network with multiple different resolution views of the mammograms^32^ (DeepHealth’s model in eFigure 9 in the Supplement).

## Discussion

The results from our study underscore the promise of using deep learning methods for enhancing the overall accuracy of mammography screening. While no single AI algorithm outperformed US community radiologist benchmarks,^2^ an ensemble of AI algorithms combined with single-radiologist assessment was associated with an improved overall mammography performance. Surprisingly, there was no additional improvement in performance when models had access to clinical variables or prior examinations. It is possible that participants did not fully exploit this information, especially the use of prior imaging examinations from the same women. This suggests that future algorithm development would do well to focus on the use of prior images from the same women to detect breast cancer. Furthermore, including additional clinical features not provided in this challenge may result in improved performance.^33^ With more than 1100 participants worldwide from 44 countries, more than 1.2 million images representing 310 827 examinations robustly linked to cancer outcomes from 2 population-based screening programs, and a third-party approach for evaluation of AI algorithms on 2 independent data sets, the DM DREAM challenge represents the most objective and rigorous study of deep learning performance for automated mammography interpretation thus far, to our knowledge.

Our trained CEM+R ensemble method used the top AI algorithms resulting from the challenge and the single-radiologist recall assessment available from the KPW training data set. When the CEM+R method was evaluated in 2 independent data sets that included single-radiologist assessments (KPW and KI evaluation sets), the ensemble method had a higher diagnostic accuracy compared with the single radiologist alone. This conclusion is consistent with a recent study demonstrating the AUC of a hybrid model that averaged the probability of malignancies estimated by a neural network and an expert radiologist outperformed the AUC of either.^17^ The improvement of the CEM+R method over the radiologist assessment was observed across all women except for the following groups: women 70 years and older in both in the KI and KPW cohorts, women with ductal carcinoma in situ, and women with at least 1 previous screening mammogram done 9 months or more earlier in the KPW cohort. In contrast, when the same ensemble method was evaluated using the consensus interpretation instead of the first radiologist assessment in the Swedish cohort, the ensemble performance did not improve in specificity. This somewhat paradoxical result is likely owing to the fact that the CEM+R ensemble was trained on the single-radiologist interpretation and thereby the importance given by the algorithm to the radiologist’s final interpretation may have been less than it would have been if the algorithm had been trained with the consensus interpretations. The performance enhancement of the CEM+R ensemble over the single-reader assessment underscores the potential value of AI as a second digital reader in a single-radiologist environment such as the United States. In the double-reading and consensus environment seen in Sweden and many other European countries, the addition of AI may not have as great an effect on improving overall diagnostic accuracy, even though it is likely that training an ensemble of AI algorithms and radiologists consensus assessments would improve over the consensus assessments alone. Taken together, our results suggest that adding AI to mammography interpretation in single-radiologist settings could yield significant performance improvements, with the potential to reduce health care system expenditures and address the recurring radiologist person-power issues experienced in population-based screening programs.

This challenge included 2 large population-based mammography data sets from 2 countries that prospectively collected consecutive screening examinations linked to clinical and longitudinal data with robust capture of breast cancer outcomes (≤12 months’ follow-up) to inform ground truth. These independent data sets differ by screening interval, cancer composition, radiologist interpretive practices, and some technical parameters (mean compression force), all of which may contribute to the algorithm performance differences between these 2 cohorts. The top-performing algorithm achieved specificities of 66.2% and 81.2% in the KPW and KI data sets, respectively, at the radiologists’ sensitivity. We believe that the reason for this difference between the 2 data sets is 2-fold. First, the 2 specificities correspond to 2 different sensitivity operating points in the 2 data sets: a sensitivity of 85.9% in the KPW data set and a sensitivity of 83.9% in the KI data set. Everything else being equal, a lower sensitivity in the KI data set will result in a higher specificity. Second, we believe the longer screening intervals in the KI data set may make the KI data set easier for cancer detection. This is supported by the difference in the AUC between the KPW and KI data sets for the top-performing algorithm (0.858 and 0.903, respectively), despite the fact that the training data set provided in the challenge consisted of an independent data set collected at KPW. Despite the important differences between these cohorts and screening programs, the performance concordance of the algorithms underscores the generalizability of our findings.

To our knowledge, this was the first study in AI and mammography benchmarking requiring teams to submit their algorithms to the challenge organizers, which permitted the evaluation of their algorithms in an unbiased and fully reproducible manner. We believe this to be an important new paradigm for data sharing and cloud-based AI algorithm development,^23^ allowing highly sensitive and restricted data such as screening mammograms to be used for public research and AI algorithm assessment. Moreover, as a stipulation of the DREAM organization and challenge funder, our fully documented algorithms are freely available to the larger research community for use and assessment in future studies of automated and semiautomated mammography interpretation.^34^

### Limitations

This study has some limitations. We recognize it is currently theoretical to combine radiologist interpretation and AI algorithms. We did not study the interaction of a human interpreter with AI algorithm results and how AI would influence radiologists’ final assessments is an area requiring greater research efforts.^5,35^ Challenge participants were unable to download and manipulate the larger training and validation image data sets, and mammography images were not strongly labeled, meaning cancer regions were not localized. We observed top-performing challenge teams using external data sets containing spatially annotated tumor information for model development had significantly higher performance in the KPW evaluation data than teams without access to strongly labeled external data (eFigure 10 in the Supplement). During the community phase with additional training data, the faster R-CNN based approach^31^ surpassed the top-performing teams’ method, which contributed to the improved performance of the ensemble model. This likely reflects that although our data sets are large, they are limited by the relatively small number of positive cases. Consequently, large comparable data sets with spatial annotation will be needed for training original algorithms or vastly larger cohorts will be required to train the next generation of AI models.

## Conclusions

In summary, the DM DREAM challenge represents the largest objective deep learning benchmarking effort in screening mammography interpretation to date. An AI algorithm combined with the single-radiologist assessment was associated with a higher overall mammography interpretive accuracy in independent screening programs compared with a single-radiologist interpretation alone. Our study suggests that a collaboration between radiologists and an ensemble algorithm may reduce the recall rate from 0.095 to 0.08, an absolute 1.5% reduction. Considering that approximately 40 million women are screened for breast cancer in the United States each year, this would result in more than half a million women annually who would not have to undergo unnecessary diagnostic work-up. Confirmation of these estimates will require additional validation and testing in clinical settings.

## Notes

Supplement. **eFigure 1. **The Timeline of the Competitive Phase of the DM Challenge **eFigure 2. **The Screening Process in Stockholm, Sweden **eFigure 3. **A Training Submission Comprises Two Docker Containers, a Preprocessing Step Followed By a Training Step **eFigure 4. **Participant Submission Workflow During the DM Challenge **eFigure 5. **Execution of Inference Submissions **eFigure 6. **Architecture of the Deep Neural Network Implemented by the Team Therapixel at the End of the Competitive Phase of the Challenge **eFigure 7. **Comparison Between a Scanned, Film Mammogram Image From DDSM Dataset (Left) and a Digital Mammogram Images From the DM Challenge Dataset Provided by KPW (right) **eFigure 8. **Outline of the Faster-RCNN Approach for Mammography **eFigure 9. **For the DREAM Challenge, Predictions Were Made on a Single-Image Basis and Averaged Across Views to Generate Breast-Level Scores **eFigure 10. **Area Under the Curve (AUC) of the Methods That Have Been Reported as A) Having Been Trained on Strongly Labelled Data (Private or Public Datasets) and B) Using an Ensemble of Models Instead of a Single Model in the Validation Phase of the Challenge **eAppendix 1. **Challenge Timeline **eAppendix 2. **Challenge Questions **eAppendix 3.** Preparation of the Challenge Datasets **eAppendix 4. **Radiologist Recall Assessment **eAppendix 5. **Challenge Datasets **eAppendix 6. **Challenge Baseline Method and Scoring **eAppendix 7.** Training and Evaluating Models in the Cloud **eAppendix 8**. Combining Model Predictions Into Ensembles **eAppendix 9**. Participation in the Challenge **eAppendix 10**. Best-Performing Models Submitted at the End of the Competitive Phase **eAppendix 11. **DM DREAM Consortium **eTable 1. **Covariates Included in the Exam Metadata File Available for Training and for Evaluation in Sub-Challenge 1 (SC1) and Sub-Challenge 2 (SC2) **eTable 2.** Mammography Views Listed in the KPW Dataset **eTable 3. **The DM Challenge Dataset Used During Leaderboard Phase **eTable 4. **The DM Challenge Dataset Used During the Validation Phase **eTable 5. **Content of the Karolinska Set in Sub-Challenge 1 and 2 Formats
eReferences.
