# Error in Author Affiliations

**DOI:** 10.1001/jamanetworkopen.2020.4429

**Published:** 2020-03-30

In the Original Investigation titled “Evaluation of Combined Artificial Intelligence and Radiologist Assessment to Interpret Screening Mammograms,”^1^ published March 2, 2020, there was an error in the Author Affiliations for Francisco Albiol, PhD, and Luis Caballero, PhD. Their affiliation should have read as follows: “Instituto de Física Corpuscular (IFIC), CSIC–Universitat de València, Valencia, Spain.” This article has been corrected.^1^
