# Supplementary material for: Evaluation of Combined Artificial Intelligence and Radiologist Assessment to Interpret Screening Mammograms
Source: JAMA Netw Open. 2020 Mar 2;3(3):e200265. doi: 10.1001/jamanetworkopen.2020.0265 (PMC7052735; doi:10.1001/jamanetworkopen.2020.0265)
Supplement: Supplement. — eFigure 1. The Timeline of the Competitive Phase of the DM Challenge eFigure 2. The Screening Process in Stockholm, Sweden eFigure 3. A Training Submission Comprises Two Docker Containers, a Preprocessing Step Followed By a Training Step eFigure 4. Participant Submission Workflow During the DM Challenge eFigure 5. Execution of Inference Submissions eFigure 6. Architecture of the Deep Neural Network Implemented by the Team Therapixel at the End of the Competitive Phase of the Challenge eFigure 7. Comparison Between a Scanned, Film Mammogram Image From DDSM Dataset (Left) and a Digital Mammogram Images From the DM Challenge Dataset Provided by KPW (right) eFigure 8. Outline of the Faster-RCNN Approach for Mammography eFigure 9. For the DREAM Challenge, Predictions Were Made on a Single-Image Basis and Averaged Across Views to Generate Breast-Level Scores eFigure 10. Area Under the Curve (AUC) of the Methods That Have Been Reported as A) Having Been Trained on Strongly Labelled Data (Private or Public Datasets) and B) Using an Ensemble of Models Instead of a Single Model in the Validation Phase of the Challenge eAppendix 1. Challenge Timeline eAppendix 2. Challenge Questions eAppendix 3. Preparation of the Challenge Datasets eAppendix 4. Radiologist Recall Assessment eAppendix 5. Challenge Datasets eAppendix 6. Challenge Baseline Method and Scoring eAppendix 7. Training and Evaluating Models in the Cloud eAppendix 8. Combining Model Predictions Into Ensembles eAppendix 9. Participation in the Challenge eAppendix 10. Best-Performing Models Submitted at the End of the Competitive Phase eAppendix 11. DM DREAM Consortium eTable 1. Covariates Included in the Exam Metadata File Available for Training and for Evaluation in Sub-Challenge 1 (SC1) and Sub-Challenge 2 (SC2) eTable 2. Mammography Views Listed in the KPW Dataset eTable 3. The DM Challenge Dataset Used During Leaderboard Phase eTable 4. The DM Challenge Dataset Used During the Validation Phase eTable 5. Content of t [file jamanetwopen-e200265-s001.pdf]

Supplementary Online Content

Schaffter T, Buist DSM, Lee CI, et al; DM DREAM Consortium. Evaluation of combined artificial intelligence and radiologist assessment to interpret screening mammograms. *JAMA Netw Open*. 2020;3(3):e200265. doi:10.1001/jamanetworkopen.2020.0265

eFigure 1. The Timeline of the Competitive Phase of the DM Challenge
eFigure 2. The Screening Process in Stockholm, Sweden
eFigure 3. A Training Submission Comprises Two Docker Containers, a Preprocessing Step Followed By a Training Step
eFigure 4. Participant Submission Workflow During the DM Challenge
eFigure 5. Execution of Inference Submissions
eFigure 6. Architecture of the Deep Neural Network Implemented by the Team Therapixel at the End of the Competitive Phase of the Challenge
eFigure 7. Comparison Between a Scanned, Film Mammogram Image From DDSM Dataset (Left) and a Digital Mammogram Images From the DM Challenge Dataset Provided by KPW (right)
eFigure 8. Outline of the Faster-RCNN Approach for Mammography
eFigure 9. For the DREAM Challenge, Predictions Were Made on a Single-Image Basis and Averaged Across Views to Generate Breast-Level Scores
eFigure 10. Area Under the Curve (AUC) of the Methods That Have Been Reported as A) Having Been Trained on Strongly Labelled Data (Private or Public Datasets) and B) Using an Ensemble of Models Instead of a Single Model in the Validation Phase of the Challenge
eAppendix 1. Challenge Timeline
eAppendix 2. Challenge Questions
eAppendix 3. Preparation of the Challenge Datasets
eAppendix 4. Radiologist Recall Assessment
eAppendix 5. Challenge Datasets
eAppendix 6. Challenge Baseline Method and Scoring
eAppendix 7. Training and Evaluating Models in the Cloud
eAppendix 8. Combining Model Predictions Into Ensembles
eAppendix 9. Participation in the Challenge
eAppendix 10. Best-Performing Models Submitted at the End of the Competitive Phase
eAppendix 11. DM DREAM Consortium
eTable 1. Covariates Included in the Exam Metadata File Available for Training and for Evaluation in Sub-Challenge 1 (SC1) and Sub-Challenge 2 (SC2)
eTable 2. Mammography Views Listed in the KPW Dataset
eTable 3. The DM Challenge Dataset Used During Leaderboard Phase
eTable 4. The DM Challenge Dataset Used During the Validation Phase
eTable 5. Content of the Karolinska Set in Sub-Challenge 1 and 2 Formats
eReferences.

This supplementary material has been provided by the authors to give readers additional information about their work.

© 2020 Schaffter T et al. *JAMA Network Open.*

eAppendix 1: Challenge Timeline

The Challenge timeline consisted of two periods: The Competitive Period, and the Collaboration Period (see **eFigure 1**).

eFigure 1. The timeline of the DM Challenge. During the Competitive Period, teams were invited to train and evaluate their models in a public leaderboard (the leaderboard phase), followed by one final evaluation in a different test dataset (the validation phase). During the Collaboration Period, teams worked collaboratively towards a final submission to the test dataset.

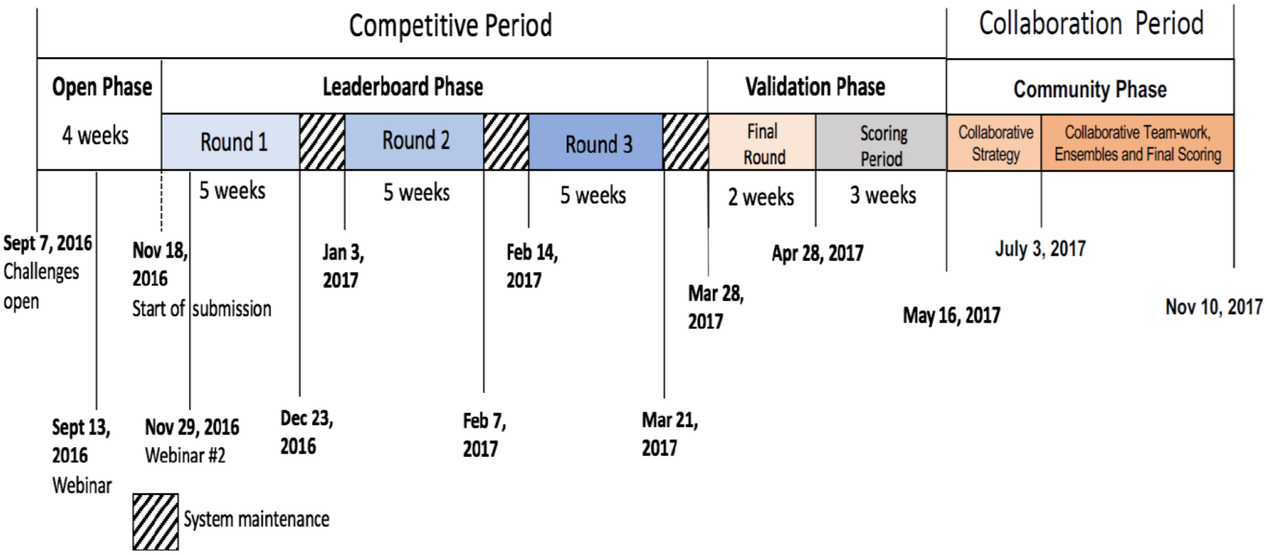

The Competitive period consisted of three phases: The Open Phase, the Leaderboard Phase and the Validation Phase, explained below:

- **Open Phase.** Ensuring that teams could train on the KPW data without being able to download the data involved innovative IT infrastructure relying on cloud computing technologies. The role of the Open Phase was to beta test the infrastructure with the help of the teams. Dummy data were used to prevent the leakage of the Challenge data during the tests. Teams also benefited from this phase by getting familiar with the infrastructure and assessing runtime characteristics of their model prior to the official launch of the Challenge.

- **Leaderboard Phase.** In this phase, teams submitted models for training and cross-validation on 50% of the KPW data. Cumulative training time per team was limited to 336 hours per Leaderboard Round, due to limited computing resources. Teams could also submit up to 3 models per round for evaluation on a hold-out leaderboard data set that represented 20% of the KPW data. Thus, teams could evaluate their models up to 9 times during the Leaderboard Phase. A total of $20,000 in cash prizes per Leaderboard Round was awarded to the top 3 performers during each of these rounds. System maintenance periods were planned between the Leaderboard Rounds and before the Validation Phase to accommodates updates and make fixes to the Challenge platform.

- **Validation Phase.** In this final phase of the Competitive Period, teams submitted their final model for scoring on a hold-out data set representing 30% of KPW data. Data from the leaderboard phase (70% of KPW data) was made available to teams to retrain their final model.

The Competitive Period was followed by the Collaboration Period, in which participants worked as a community towards the final submission to the evaluation datasets.

eAppendix 2. Challenge Questions

The goal of the DM Challenge was to develop models that analyze screening mammography exams and interpret them as to whether the subject has breast cancer or not, and, if so, in which breast. For each breast, a screening mammography exam can be positive or negative, operationally defined as follows:

- **Positive breast.** The cancer status of a given screening mammography exam was defined as positive in the left/right breast if the subject was diagnosed with breast cancer in the left/right breast (confirmed with tissue diagnosis) within 12 months of the given screening mammography exam. The tissue diagnosis may have been prompted by a call-back due to findings on the current screening mammography exam, a short-interval follow-up examination, or other clinical exams.

- **Negative breast.** The cancer status of a given screening mammography exam was defined as negative in the left/right breast if the subject did not have a known diagnosis of cancer in the left/right breast on review of medical records one or more years after the screening exam.

We also wanted to identify whether using clinical and/or longitudinal data in addition to the current mammography images could increase the performance of the models. This led to the creation of two Sub-challenges, embodying the main questions asked in the Challenge:

- **Sub-challenge 1**
    - **Definition.** Determine the cancer status of each breast of a subject, given only a screening digital mammography exam (without access to previous exams or clinical/demographic information).
    - **Input.** A screening mammography exam consisting of several images of both breasts.
    - **Output.** Two scores (SL, SR), each between 0 and 1, indicating the likelihood that the subject was tissue-diagnosed with cancer within one year from the given screening exam, in the left (L) and right (R) breast respectively.
- **Sub-challenge 2**
    - **Definition.** Determine the cancer status of each breast of a subject, given a screening exam, a panel of clinical/demographic information, and if available, previous screening exam(s).
    - **Input.** The given exam consisting of several images of both breasts and, if available, previous screening exams of the same subject, clinical/demographic information such as race, age and family history of breast cancer.

- **Output.** Two scores (SL, SR), each between 0 and 1, indicating the likelihood that the subject was tissue-diagnosed with cancer within one year from the given screening exam, in the left (L) and right (R) breast respectively.

Teams could choose to participate in either one or both Sub-challenges. In Sub-challenge 1, only one mammography exam was given for a subject. The inference of cancer/non-cancer in each breast had to be based on that mammography exam images only (without access to previous exams or clinical/demographic information). In Sub-challenge 2, one or more screening mammography exams were given for a subject along with metadata (clinical and demographic) information. If there was one exam, then the inference had to be made based on that exam and the metadata. If there was more than one exam in the longitudinal series, then the cancer status of each breast was asked for only the most recent exam. All previous exams correspond to negative exams both in left and right breasts.

When submitted for evaluation, we asked that models process subjects individually and generate a confidence level in the interval [0,1] for both breasts. In other words, the confidence levels for a subject should be independent of the other subjects in the evaluation data set. However, for a given subject, it was fine to have the confidence level of one breast depending on the data from the other breast.

eAppendix 3. Preparation of the Challenge datasets

Here we describe the protocol that we have applied to refine the original data received by Kaiser Permanente Washington (KPW) to generate the training and evaluation sets for the DM Challenge. First, we explored the original data and fixed inconsistencies. Then, we undertook an analysis of potentially predictive variables and tailored the dataset to prevent data leakage, which can lead to poor generalization and over-estimation of a model's performance[1]. Finally, we describe how we have split the training and evaluation sets for the DM Challenge Sub-challenge 1 and 2 so that each set was representative of the global population of subjects.

Refinement of the Kaiser Permanente dataset

KPW uploaded 640,905 de-identified mammogram images in DICOM format (14.1 TB) to an AWS S3 bucket managed by Sage Bionetworks. The integrity of the images transferred was verified using MD5 checksum. The images were also transferred to a secured cloud provided by IBM where the exploration and refinement of the data took place. KPW also provided two files that included information about 146,371 mammography exams for 86,873 women.

1. **Exam metadata file.** This file provided clinical/demographic information for each exam included in the dataset such as subject ID, exam index, time since last exam, subject age, and patient-reported data on first degree family history of breast cancer, body mass index (BMI), etc. A comprehensive description of clinical/demographic information available to the algorithms during the Challenge is given in **Table S1**.

2. **Image crosswalk file.** This file linked individual images to their respective exam. This file also provided information about laterality (left or right) and view (CC, MLO, etc.) used to image the breast (see **Table S2**).

Before starting to explore the content of these files, we extracted the information available in the header of the DICOM images. The DICOM header includes information about the scanner (e.g. scanner manufacturer, model, software version, etc.) and the conditions in which the breast was imaged (radiation

dose, exposition time, compression force, etc.). While extracting the content of the DICOM header, we only found 3 corrupted images, which were subsequently removed from the dataset.

The DICOM header also include deidentified clinical information. The content of the metadata files and DICOM headers had been previously obfuscated by KPW in order to prevent the identification of individual subjects in compliance with the Health Insurance Portability and Accountability Act (HIPAA), which sets the standard for protecting sensitive patient data in the US.

Below we list the modifications applied to improve the consistency of the dataset.

- **Removing exams without images.** There were a few exams listed in the exam metadata file for which no images were provided. We carefully removed these exams, as data from one exam depended on the data from the previous exam (exam index, number of days since the previous exam).

- **Fixing inconsistent laterality and views.** We compared the breast laterality (left or right) and the view (CC, MLO, etc.) specified for each image in the image crosswalk file with the value specified in the DICOM headers. We found a dozen images for which either the laterality and/or view were not matching. The inconsistencies were fixed either in the image crosswalk file or in the DICOM header after visualizing the images. We keep both sources of laterality and view for two reasons. First, these two values were expected to be found in the DICOM header by existing software[2]. Second, opening all the image files to figure out this information is a time-consuming task considering the amount of images available during the DM Challenge. This is particularly true for methods that work only on CC images, for example. With more than 1000 registered participants before the launch of the Challenge and limited computational power, we decided to provide this information in the image crosswalk file to help teams saving their allotted computational time, which can then be used for more meaningful calculations.

Preventing information leakage

In Machine Learning, information leakage occurs when a predictive model is trained using information about the desired prediction that is, perhaps unintentionally, included in the training set but not available to the model when making predictions for unseen data. Models experiencing information leakage tend to be very accurate during development, but perform poorly when making predictions for de novo subjects [1,3].

To prevent leakage, we undertook a proactive analysis of potentially predictive variables and carefully considered which ones to make available to the training models. This analysis was performed using all the covariate available, including information from the DICOM header of the images. Here, we list below two potential sources of information leakage that we identified and that could have affected the performance of the predictive methods when applied to new datasets.

- **Removing clinical information from the DICOM header in Sub-challenge 1.** The goal of Sub-challenge 1 was to develop methods that make predictions based solely on the content of the images (pixel values and technical information from the DICOM header) without having access to clinical/demographic information.

- **Removing subjects who have at least one exam with one breast not imaged.** There were 1073 subjects (1.2% of all the subjects) who had at least one exam where there were no images

© 2020 Schaffter T et al. *JAMA Network Open.*

for the left or right breast. The fraction of positive subjects with at least one exam "missing" (3% of positive subjects) was significantly larger than for negative subjects (Fisher's exact test: $p<0.05$). These subjects were removed to prevent predictive methods to associate the occurrence of missing breasts with breast cancers.

- **Removing XCCL images.** A laterally exaggerated CC (XCCL) view is a supplementary mammographic view performed when tissue extends to the edge of the field of view on the CC view and tissue projects on the pectoral muscle on the MLO view. An XCCL view is also done when a lesion is suspected on a MLO view but cannot be seen on the CC view. We found that the fraction of positive exams with laterally exaggerated CC (XCCL) views (8.2% of positive exams) in KPW dataset was significantly larger than the fraction of negative exams with XCCL views (4.5% of negative exams, Fisher's exact test: $p<0.05$). In order to prevent methods to use the presence of XCCL images as a predictive feature while trying to conserve as many images as possible, we decided to randomly pick positive exams and remove their XCCL images until the difference is no longer significant when comparing to negative exams ($p > 0.05$).

Selecting covariates for training and evaluation sets

Here, we describe the covariates that we have included in the training set of the DM Challenge and in the evaluation sets of Sub-challenge 1 and 2.

The exam metadata file provided by KPW included clinical and demographic information, radiologist assessment and biopsy results (when a breast biopsy occurred ≤12 months from screening mammogram). In order to enable a fair comparison of the performance of radiologists and predictive methods, we decided to not make available information generated by the interpreting radiologist (**eTable 1**). Therefore, the recall assessment of the radiologist was not made available. For the same reason, we decided not to include the breast density estimated by the radiologist. Since breast cancer originates in epithelial cells from dense, fibroglandular tissue[4], we hope that this decision helped make participants consider unsupervised alternatives to estimate breast density[2].

© 2020 Schaffter T et al. *JAMA Network Open.*

eTable 1. This table describes the covariates included in the exam metadata file available for training and for evaluation in Sub-challenge 1 (SC1) and Sub-challenge 2 (SC2). Regarding the training set, we decided to provide a single dataset that included both longitudinal and clinical/demographic information about the training subjects.

Name	Description	Values	Training	SC1 scoring	SC2 scoring	Comments
subjectId	Subject ID		Yes	No	Yes	Unique per subject
examIndex	Index of the exam for given subject		Yes	No	Yes	Set to 1 for the first exam of the subject
daysSincePreviousExam	Number of days since the previous screening exam		Yes	No	Yes	Set to 0 for the first exam
cancerL	Whether the left breast developed a cancer w/in 12 months	0 = No<br>1 = Yes<br>. = Breast nor imaged	Yes	No	But exam to predict	
cancerR	Whether the right breast developed a cancer w/in 12 months	0 = No<br>1 = Yes<br>. = Breast nor imaged	Yes	No	But exam to predict	
invL	Whether the cancer in the left breast was invasive or not (DCIS)	0 = No<br>1 = Yes<br>. = Breast nor imaged	Yes	No	But exam to predict	Invasive cancer diagnosed within one year. Using SEER and breast pathology data.
invR	Whether the cancer in the right breast was invasive or not (DCIS)	0 = No<br>1 = Yes<br>. = Breast nor imaged	Yes	No	But exam to predict	Invasive cancer diagnosed within one year. Using SEER and breast pathology data.
age	Age at screening exam		Yes	No	Yes	
implantEver	Ever had implants	0 = Never<br>1 = Ever had implants<br>. = Unknown/missing	Yes	No	Yes	Using self-report and implant removal procedure code in pathology data.
implantNow	Laterality of the implant(s) in place today (if any)	1 = Right Breast only<br>2 = Left Breast only<br>4 = Bilateral<br>5 = Yes, woman-level info only<br>. = Unknown/Missing	Yes	No	Yes	
bcHistory	Personal history of breast cancer	0 = None<br>1 = Yes	Yes	No	Yes	Previous diagnosis collected from previous SEER registry, breast pathology and from self-report on the date of the exam.
yearsSincePreviousBc	Years since prior breast cancer diagnosis		Yes	No	Yes	
previousBcLaterality	Laterality of prior breast cancer	1 = Right Breast only<br>2 = Left Breast only<br>3 = Unspecified laterality<br>4 = Bilateral	Yes	No	Yes	
reduxHistory	History of breast reduction	0 = None<br>1 = Prior breast reduction<br>. = No history of breast reduction found	Yes	No	Yes	Using both self-report and pathology breast reduction procedures dated before this screening mammography exam.
reduxLaterality	Laterality of breast reduction	1 = Right breast only<br>2 = Left breast only<br>4 = Bilateral<br>. = No history of breast reduction found	Yes	No	Yes	
hrt	Current use of Hormone Replacement Therapy (HRT)	0 = No<br>1 = Yes<br>9 = Unknown	Yes	No	Yes	Self-report only
antiestrogen	Current use of anti-estrogen therapy (Tamoxifen/raloxifene)	0 = No<br>1 = Yes<br>9 = Unknown	Yes	No	Yes	Self-report only
firstDegreeWithBc	First degree relative with BC	0 = No<br>1 = Yes<br>9 = Unknown	Yes	No	Yes	Self-report only
firstDegreeWithBc50	First degree relative with BC < 50 years old	0 = No<br>1 = Yes<br>9 = Unknown	Yes	No	Yes	Self-report only
bmi	BMI at screening exam		Yes	No	Yes	BCSC computes BMI with the Imperial formula: (703.069*weight/(height**2)) Using self-report height and weight, restricting height to 48" - 87", weight to 50 - 500 lbs, allowing BMI values of 15 - 90. When weight is missing from current exam self-report, search records within one year before and after. When height is missing from current exam self-report, search through all records, no time restriction, for valid value closest in time.
race	Race, NIH reporting standard	1 = White<br>2 = Black<br>3 = Asian<br>4 = Hawaiian/Pacific Islander<br>5 = American Indian/Alaska Native<br>6 = Other<br>7 = Mixed<br>8 = Hispanic<br>9 = Missing	Yes	No	Yes	Self-report only

© 2020 Schaffter T et al. *JAMA Network Open.*

Splitting KPW data into training and evaluation sets

To maximize generalizability and minimize strata bias, models were trained and evaluated on data representative of the true population's distribution. This was achieved by implementing a probability sampling technique known as proportionate stratified random sampling[5] to partition the KPW dataset into 50% training set, 20% evaluation for the Leaderboard Phase and 30% evaluation set for the Validation Phase. First, the dataset was multi-level sorted in ascending order of variable class variance (i.e. *cancer* > *invasive* > *bilateral breast cancer* > *ethnicity* > *age* > *BMI*). We then sequentially iterated over the sorted records and proportionally distributed them into three subsets using an *ad hoc* cup and bucket algorithm. Three cups of sizes 2, 3 and 5 records, corresponding to 20%, 30% and 50%, respectively, were initialized, and a token was placed in the first cup. For each record along the sequence, if the cup carrying the token was not full and the subject the record belonged to was not in other cups or buckets then the record was added to the cup. Otherwise, if the cup was full but the record belonged in this cup then the record was added straight to the corresponding bucket, before summoning the next cup to grab the next record. Once the largest cup (50%) was full, all cups were emptied into their respective buckets and the process was repeated until all records were exhausted. Consequently, this algorithm prevented subjects from appearing in multiple subsets, which was a Challenge requirement. We then compared statistics across the three subsets to assess distribution similarity. Finally, 10 sets of training, Leaderboard evaluation and Validation evaluation sets were generated with inter-set stochastic variance (random-shuffled records within strata before sampling) to identify the median dataset that was eventually used in the Challenge.

Pilot Set

In the DM Challenge, participants were not allowed to download or directly access the Challenge training or validation datasets. For this reason, we developed an approach called Model to Data that we expect to pave the way for future competitions that will make use of datasets that cannot be made public (e.g. medical information, data from a company that want to crowdsource a problem, etc.). The details of the infrastructure is described in **Section Training and evaluating models in the cloud**.

To provide insights into the data of this Challenge, we were authorized by KPW to release a small dataset called the DM Challenge Pilot Data. This set included 500 mammography images as well as the clinical information of 58 cancer positive and negative subjects. The goal was not to provide a small set that was representative of the population of the global dataset but rather to share the specifications of the images and show examples of exams correctly and incorrectly classified by radiologists.

Moreover, subjects were selected to include examples of visible artifacts that we expected could distract predictive methods. Examples of artifacts included in the Pilot Set were skin markers to identify nipples, moles, scars, the edges of the compression paddle, surgical clips from prior excision, lumpectomy and central line/port for iv medication infusion, etc.

DICOM images

We received the DICOM images from Kaiser Permanente in compressed format (.dcm.gz) for a total of 3.8 TB. Uncompressed, the DICOM images take 14.1 TB. We decided to provide the images uncompressed to the predictive methods because the time required to uncompress them can then be saved by the predictive methods for more meaningful calculation. This comes at the expense of higher cost to host the data, especially in a configuration where multiple copies of the dataset are instantiated in the cloud to ensure that methods can read the images with enough bandwidth.

eTable 2. Mammography views listed in the KPW dataset. During a routine screening mammography, each breast is usually imaged on at least one CC and one MLO views (standard views). Additional CC/MLO views or other types of views are sometimes performed by the radiologist, for example when additional images are required to capture the entirety of the breast.

View	Description
AT	axillary tail
CC	craniocaudal
CCID	craniocaudal (implant displaced)
CV	cleavage
FB	from below
LM	90° lateromedial
LMO	lateromedial oblique
ML	90° mediolateral
MLID	90° mediolateral (implant displaced)
MLO	mediolateral oblique
MLOID	mediolateral oblique (implant displaced)
RL	rolled lateral
RM	rolled medial
SIO	superior inferior oblique
XCCL	exaggerated craniocaudal lateral
XCCM	exaggerated craniocaudal medial

eAppendix 4. Radiologist recall assessment

Kaiser Permanente Washington

KPWA breast cancer screening guidelines follow USPSTF recommendations, which include risk assessment and recommended shared decision making for women aged 40-49 and annual or biennial screening for women aged 50-74 years aligned with a woman's personal risk factors[6]. All women can choose to undergo annual screening mammograms with no cost, regardless of their risk.

All screening mammography exams are interpreted by a single radiologist, using ACR's BI-RADS assessments[7]:
0: incomplete (need additional imaging evaluation)
1: negative
2: benign findings
3: probably benign

© 2020 Schaffter T et al. *JAMA Network Open.*

4: suspicious abnormality
5: highly suspicious of malignancy
6: known biopsy with proven malignancy

Unlike in the Karolinska population, double reading is not standard in the US. Using standard US definitions[8], we created an initial overall assessment for the screening examination, using the most serious BI-RADS Breast Imaging Reporting and Data System assessment according to the following hierarchy: negative, 1; benign, 2; probably benign, 3; needs additional evaluation, 0; suspicious, 4; and highly suggestive of malignancy, 5. Each screening examination was followed for 12 months after a screening mammogram (truncated at the next screening mammogram to ensure only one cancer was linked to each screening exam).We included all SEER and BCSC pathology endpoints with a diagnosis of invasive breast carcinoma or DCIS.

Using BI-RADS definitions, women who receive an initial BI-RADS assessment of 0, 3, 4, 5, or 6 are considered to be recalled from screening (positive recalls)[7]. One-year cancer follow-up was determined by linkage with regional cancer registries to establish true-positive, true-negative, false-positive, and false-negative screening results (gold standard).

Initial assessments were linked with cancer outcomes to define screen-detected vs. false-positive outcomes. Women that were not recalled but later had a clinically detected cancer before their next scheduled screening mammogram (an interval cancer) were classified as having false-negative screening exams.

Karolinska Institute

The current national recommendation in Sweden stipulates that women should be invited for screening starting at age 40 and ending at age 74. All women fulfilling the age criteria are invited to screening, and they will continue to receive invitations whether they choose to attend or not. The national recommendation further stipulates that the time interval between invitations for screening should be between 18 and 24 months, with the shorter time interval suggested for younger women. The participation rate in Stockholm exceeds 70%[9].

© 2020 Schaffter T et al. *JAMA Network Open.*

eFigure 2. The screening process in Stockholm, Sweden. The process until recall decision is shown in the top row. Around 97% are assessed to lack signs of malignancy, while around 3% are recalled for further assessments according to the bottom row. Around 0.5% are diagnosed with breast cancer.

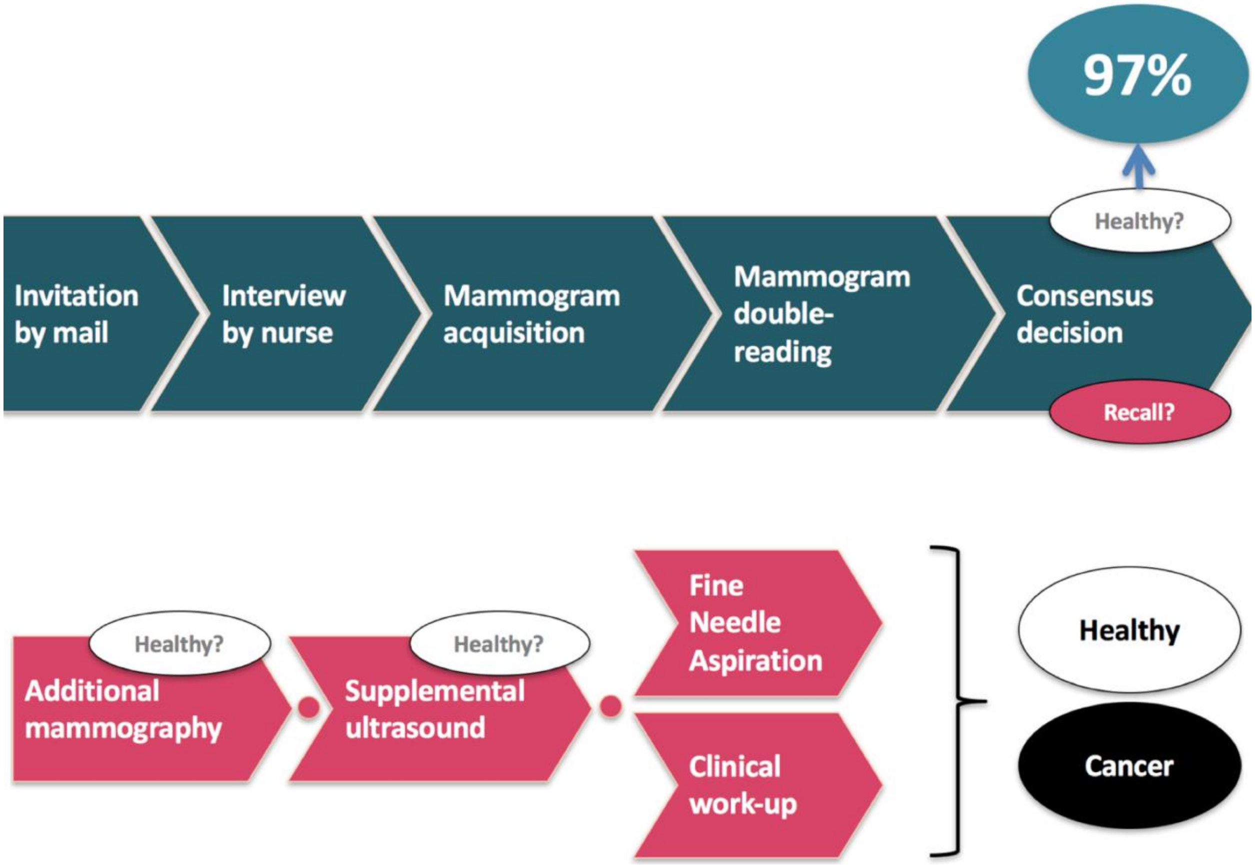

The screening process is described in e**Figure 2**. Double reading means that two radiologists independently assess each case and identify suspicious findings which are ‘flagged’ for consensus discussion.

For each diagnosed breast cancer, the ‘detection mode’ was categorized as: screen-detected cancer (SDC), interval cancer (IC) or ‘non-attender’ (women who did not attend the prior screening). In a pooled analysis of six European countries, including Sweden, published in 2010, IC constituted 28% of cancers[10]. The IC rate for the first 12 months after screening was 5.9 per 10,000 women and another 12.6 per 10,000 women the following 12 months. For Stockholm, the recall rate was 3.3% for initial screening and 1.8% for subsequent screenings; among screened women 69% of cancers were screen-detected and 31% were interval cancers. According to a review of several interval cancer studies the proportions are generally between 17% and 30% for biannual screening[11]. In one study of annual screening it was 15%, and in a few studies of 3-year intervals it was 32% to 38%.

© 2020 Schaffter T et al. *JAMA Network Open.*

eAppendix 5. Challenge datasets

Kaiser Permanente Washington (KPW) dataset

The following tables summarize the number women, exams and images used during the Challenge Leaderboard Phase and Validation Phase (see **Section Challenge Timeline**).

eTable 3. The DM Challenge dataset used during Leaderboard Phase. The training set included 50% of the women in the KP dataset. The subjects in Sub-challenge 1 and 2 were the same and represent 20% of the KP dataset.

	LEADERBOARD PHASE								
	Training			Sub-challenge 1			Sub-challenge 2		
	Positive	Negative	Total	Positive	Negative	Total	Positive	Negative	Total
Women	481	42,336	42,817	188	16,918	17,106	188	16,918	17,106
Exams	481	71,652	72,133	188	16,918	17,106	188	28,653	28,841
Images	1,114	316,503	317,617	434	75,428	75,862	434	126,338	126,772

eTable 4. The DM Challenge dataset used during the Validation Phase. The training set included the training and Sub-challenge 2 evaluation sets used during the Leaderboard Phase (70% of the KP dataset). The women in Sub-challenge 1 and 2 were the same and represent 30% of the KP dataset.

	VALIDATION PHASE								
	Training			Sub-challenge 1			Sub-challenge 2		
	Positive	Negative	Total	Positive	Negative	Total	Positive	Negative	Total
Women	669	59,254	59,923	283	25,374	25,657	283	25,374	25,657
Exams	669	100,305	100,974	283	25,374	25,657	283	42,974	43,257
Images	1,548	442,841	444,389	646	113,470	114,116	646	189,894	190,540

We were eager to identify other datasets that could be used to provide independent validation from the KPW data. We collaborated with the Karolinska Institute, Sweden, which brought in a dataset similar in size of the one provided by Kaiser Permanente Washington (> 600k images). Here, we refer to this dataset as the Karolinska dataset. One condition set by the Karolinska Institute was that the dataset must only be used for the purpose of evaluating the performance of predictive models and not to train them.

We applied the same protocol as described in **Section Preparation of the Challenge datasets** and **Preventing information leakage** to curate and format the Karolinska dataset in the same fashion as we prepare the Challenge (KPW) dataset. **eTable 5** describes the content of the Karolinska dataset in Sub-challenge 1 and 2 format.

eTable 5. Content of the Karolinska set in Sub-challenge 1 and 2 formats.

	KAROLINSKA SET					
	Sub-challenge 1			Sub-challenge 2		
	Positive	Negative	Total	Positive	Negative	Total
Women	784	67,242	68,026	784	67,242	68,026
Exams	784	67,242	68,026	784	165,812	166,596
Images	1,557	266,632	268,189	1,557	656,740	658,297

The content of the Karolinska set differed from the KPW dataset on several points that will be introduced and discussed in the following sections. Besides a few covariates such as the age of the women and the time since the last mammography exam, which are described in the next section, the Karolinska dataset did not include extensive clinical/demographic information as was available in the KPW dataset. A second difference is that the Karolinska set provides only one CC and one MLO view per breast (standard views) while the KPW dataset provides a more diversified collection of views (see e**Table 2**) along with often multiple instances of the same view.

Optimam dataset

The location of lesions was not available for the Challenge dataset (weakly-labeled data). After discussing with the best performers of the DM Challenge in preparation for the community phase, it appeared that most of the teams had pre-trained their methods on either public or private strongly labeled (location of abnormalities available) data before training on the Challenge dataset. Examples of public, strongly labeled dataset include as the Digital Database for Screening Mammography (DDSM)[12], the INBreast dataset[13] and the Mammographic Imaging Analysis Society (MIAS)[14].

The team London Mammo offered to share with the other participants to the community phase the strongly-labeled dataset that they have used during the competitive phase. The Optimam Image database,[15] which has images from the UK National Health Service Breast Screening Programme (NHSBSP). The NHSBSP offers screening mammography to women aged between 50 to 70. However, some women in the age ranges 47-49 and 71-73 had screening as part of a trial to evaluate the effectiveness of extending the UK screening age range. The database has collected prospectively since 2011 screening mammograms of all breast cancers detected at three screening centers in the NHSBSP. Representative samples of screening mammograms of women who attended screening but were not recalled for further investigation are also included in the database. The subset of the Optimam image database provided for the DM DREAM Challenge contained 4,500 cases made up of 3,500 malignant and benign cases and 1,000 normal. Each case has from 1 to 52 screening and symptomatic images for a total of 78,377 images. Most images are Full Field Digital Mammograms while some are magnification

© 2020 Schaffter T et al. *JAMA Network Open.*

images used in diagnosis and generally centered on a suspected lesion which have an estimated radiographic magnification factor above 1 and up to 1.8 times. 52.7% of the images have been processed by the device manufacturer's algorithms for presentation to radiologists, while 47.3% of images are raw device output. Associated metadata includes image-related information (DICOM header and expert annotations) as well as clinical observations. They were collected by three hospitals in the UK and Belgium mainly from Hologic and GE devices. Image sizes ranged from 512 x 512 to 4,915 x 5,355 pixels. A subset of 7,500 images had annotated findings. Annotations are rectangular bounding boxes around anomalous tissue, making this dataset strongly labelled.

eAppendix 6. Challenge baseline method and scoring

Challenge baseline methods

We developed the Challenge baseline methods for Sub-challenge 1 and 2 during the preparation of the Challenge. In order to be eligible for the cash prize, participants were requested to outperform the performance of the baseline method in the Sub-challenge of interest. The development of these baseline methods also helped to identify the computational resources (RAM and GPU memory, scratch space, etc.) that participants would need to answer the Challenge questions. Using popular deep learning frameworks such as Caffe and TensorFlow, we were also able to evaluate the runtime quota that we allotted to each participating team.

Evaluation metrics

In the competitive phase of the Challenge, participants were asked to determine the cancer status of each breast of a subject (see Challenge Questions section) and the performance was evaluated at the breast-level. This means that for each woman, algorithms had to output two scores indicative of the likelihood that each breast was diagnosed with cancer within a year. Therefore, each breast was considered independently for scoring. The primary metric used for performance assessment was the area (AUC) under the receiver operating characteristic curve (ROC). The AUC can be thought of as the average value of sensitivity over all possible values of specificity and is a measure of how well the algorithm's continuous score separates positive from negative breast cancer status. It is considered to be one of the major metrics for the assessment of computer-aided diagnosis algorithms[16,17]. In this first phase of the Challenge, we did not want to tie the evaluation of different algorithms with encouraging results to the performance at a fixed sensitivity or specificity (i.e., operating point), but we wanted to identify algorithms that perform well across a range of operating points that might be combined in a synergistic way in the collaborative phase. A secondary metric, used only for tie-breaking between algorithms, was the partial area under the ROC curve (pAUC) above a sensitivity of 0.82. Our protocol stipulated that after ranking submissions with respect to AUC, ranking robustness tests would be performed between the high-ranking algorithms. If the highest-ranked algorithm was robustly better than the second highest, then the top algorithm was the winner of the Sub-challenge. Robustness was assessed using a bootstrapping-based calculation of the Bayes factor[18], which is equivalent to inverting a bootstrap percentile confidence interval using a significance level equal to 0.05. If the top algorithm was not robustly better than the second, then the third, fourth, etc. algorithms would also be tested to define a group of algorithms that is tied in robustness, and within this group, the secondary metric (pAUC) would be used to re-rank the tied algorithms.

In the collaborative phase and in all the results reported in the main paper, algorithms were required to output the risk that the woman had cancer independent of the breast affected; that is, algorithms were

© 2020 Schaffter T et al. *JAMA Network Open.*

scored at the woman-level, or given that there were women with more than one exam, at the exam-level. Interestingly, for the same algorithm, performance metrics such as AUC and specificity at the exam level were worse than at the breast level. Scoring at the exam level was necessary because we wanted to tie the Challenge assessment metric to the mean performance of radiologists interpreting digital screening mammograms, whose performance is evaluated at the exam level[7]. A recent study with over 1.5 million digital mammograms from 2007 to 2013 found that in screening, the mean sensitivity and specificity of radiologists, calculated following American College of Radiology BI-RADS definitions, were 0.87 and 0.89, respectively[19]. The goal of the collaborative phase was to design an algorithm that approached this target performance, although, as discussed in more detail below, due to the asymmetry in the way radiologists and algorithm are evaluated, this might be a high target for the algorithm to achieve. To measure the closeness of the algorithm to the target performance, we set a target sensitivity of 0.87 for the algorithm and focused on the specificity achieved by the algorithm. The participants in the collaborative phase were rewarded based on the improvement upon the best specificity achieved in the Challenge phase (specificity=0.65) at this sensitivity, with the full prize awarded if they could reach the mean radiologist specificity of 0.89.

eAppendix 7. Training and evaluating models in the cloud

Technical constraints

The compute set-up for the Challenge reflected several constraints, which we first describe.

Isolation of Training Data: Typically, machine learning Challenges allow participants to download a training data set along with the input side of the inference data, to their own machine, then submit their predictions to be scored against withheld validation data. However, in this Challenge the data donor required that sample dataset downloaded by participants not exceed a tiny sample set (500 images) and that the full dataset not be accessible via a participant managed endpoint (either locally or cloud-based) at all. Additional factors discouraged the practice of having participants download data. For example, the sheer size of the data (tens of terabytes) suggested that it would be better to move models to the location of the data rather than the reverse. We expected that models would require high-end GPU processors for effective training. If we required participants to purchase or otherwise gain access to such compute power, it might have biased the Challenge in favor of those with greater resources. Using a common compute environment, provided by the Challenge organizers, would help to create a level playing field.

Incomplete information about participants and compute: A primary goal for organizing an open Challenge is to attract a broad segment of the community. This makes it hard to predict the actual number of participants. While we can use past participation as a guideline, the high profile and cash awards of this Challenge meant that there might be much greater participation than usual. While past Challenges had 100 or so active participants, there were over 1000 pre-registered for this Challenge. This suggested a high level of interest but did not give a precise level of expected participation. We needed a technical solution that would both allow us to control the level of resources consumed by each participating team and that would allow us to scale as participation grew. We had the benefit of two donors of cloud compute, IBM Cloud and Amazon Web Services, from which we could provision resources. We needed an architecture that was cloud agnostic and scalable, allowing us to quickly add more machines when required.

Performance: We needed to acquire high performance GPU servers and to mount the data such that it could quickly be moved into the GPU processors' memory for model training.

© 2020 Schaffter T et al. *JAMA Network Open.*

Reuse: We wished to ensure the models would be able to be rerun in the future, on commodity hardware, perhaps not identical to that used in the challenge.

Early in the challenge two additional requirements became apparent:

Reuse of preprocessed data: Model training by participants typically included two phases: First, the data might be preprocessed in several ways; for example, conversion from the native DICOM medical image format to another (JPG, PNG), scaling, or cropping. Training was subsequently performed on this preprocessed data. The first step could take several days to complete but was generally not then changed. The second step was frequently altered, as participants tried variations of parameters and algorithms. To be productive there was a clear need to *cache* the preprocessed data and to use it for multiple machine learning iterations.

Checking for errors: Before opening the challenge, we had hoped that participants could try their models on the small pilot data set so that when they eventually submitted their model to train against the full data set all bugs would have been worked out. In practice there were unforeseen problems, e.g., due to variations of data not in the pilot set, or due to the way that the challenge infrastructure merged the models and data for training. The result was that models might run for days before an unrecoverable error was detected. A mechanism was needed to quickly reveal problems with candidate models.

Infrastructure Design

The various constraints described above suggested the use of a batch submission system, with limits imposed on participants. The items submitted would be models to be run. The series of DREAM challenges has long made use of the Synapse web-based platform for scientific collaboration[20]. Synapse provides data sharing, wiki-based project descriptions and discussion forums, among other features. It includes submission queues, originally intended for submitting predictions (files) for scoring. For this challenge we used the submission queues to submit *models* for execution by the batch processing queue. Participants were asked to structure their trainable model into two steps, (1) preprocessing and (2) training. Moreover, each step was to be created in the form of a lightweight portable machine image called a Docker container[21]. Docker images are stored in what are called Docker registries. Public registries include DockerHub[22] and Quay[23]. To support the challenge, we added a Docker registry to Synapse itself. This ensured that participants would not encounter fees or other constraints when storing their models. The entries in a Docker registry are called Docker repositories. Each repository is a series of versions called commits and each commit is akin to an image of a Unix file system. Commits are referenced by user defined tags (names) or by unique, system defined IDs. A typical Docker repository name in Synapse is:

docker.synapse.org/syn4224222/dm-caffe-training-example

Where 'docker.synapse.org' refers to the Synapse Docker registry, 'syn42224222' refers to a Synapse project (in this case the Digital Mammography project) and 'dm-caffe-training-example' is a user defined name. A specific version is referenced by its system generated commit, a SHA-256 hash, e.g.,

Sha256:eadc45274677cc12f1f61570bbac390cc4937e9c9f73782d40c84e89784a0cd5

The submission to a challenge for training is a pair of such references:

preprocessing=docker.synapse.org/syn4224222/dm-preprocess-caffe@sha256:84e9...6341

training=docker.synapse.org/syn4224222/dm-train-caffe@sha256:c129...ff44

Where the first in the pair is the image of the containerized code for doing preprocessing and the second is that for performing the subsequent machine learning. If a later submission combined an existing preprocessing step with a modified training algorithm the first line would be the same while the second would either reference a new repository or a new version of an existing one. The use of specific version references in submissions prevented *race conditions*, where otherwise a participant might modify a repository after submission, causing the executed submission to be different from the code at the time of submission. Requiring the participants to specify the preprocessing *algorithm* rather than the preprocessing *output* had several advantages:

- Freed us as challenge administrators from having to preserve preprocessed data created on ephemeral storage;
- Ensured against loss of unrecoverable information if preprocessed data were lost;
- Allowed organizers to freely reconfigure back end resources;
- Provided reproducibility of each submission

Several participants requested the ability to make quick, small modifications to existing preprocessed data rather than prolonged reprocessing from scratch to incorporate small preprocessing changes. These requests were denied since allowing them would negate the advantages described above.

An alternative to using Docker would be to allow submission of a compiled executable. A big advantage of the Docker-based approach is that a Docker image has its software dependencies bundled together and therefore is highly portable. A participant can create and test out their container locally, then submit it for processing, confident that it will work the same on the challenge infrastructure as it does on their own system. This approach not only allows automatic execution of submitted code but also helps with reproducibility, the latter being fundamental to ensuring the validity of any resulting scientific discovery. Each submission contains user applications, language runtimes, packages and libraries, as well as the machine learning framework used by the participant. We found that the most popular framework used by participants was Caffe[24]. However, many participants also used other frameworks such as TensorFlow[25], Theano[26], and MXNet[27]. We provided sample Docker images for multiple frameworks.

A feature of Docker is the ability to mount host volumes (folders) to be accessible within the running container. The challenge instructions specified the location of the data as well as an empty folder into which the model under training would maintain its state. At the end of training the contents of the model state folder were zipped and uploaded to the Synapse data sharing platform. The flow is summarized in **eFigure 3**.

eFigure 3. A training submission comprises two Docker containers, a preprocessing step followed by a training step. Preprocessing takes as input the raw image data and image metadata, writing output to 10 TB ephemeral storage (the preprocessing cache). The training step reads from the preprocessing cache and maintains its state in the model state folder. 500 GB of scratch space is also made available. During preprocessing and training, logs are captured and returned to the submitter. At the end of training, the model state is stored for retrieval by the submitter.

© 2020 Schaffter T et al. *JAMA Network Open.*

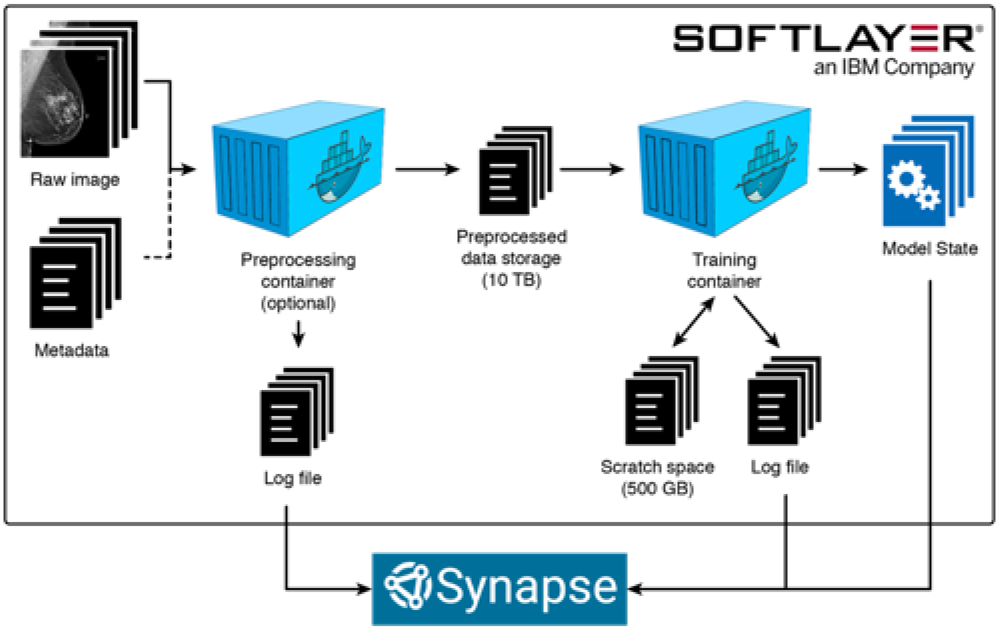

By tracking the submitting team and the preprocessing Docker image used to generate each set of cached preprocessed files, the system could reuse the cached intermediate result. If a team chose to modify their preprocessing algorithm, their cache was overwritten with the new content.

The overall flow of the Challenge is shown in **eFigure 4**. Participants started by visiting the Synapse web site to learn about the Challenge and register. Once registered, they had access to Challenge data, example models, and GitHub links to source code. After developing a candidate model, participants pushed the Docker image to Synapse. Once a model started running, an email was sent with a link for retrieving log files. Participants could monitor progress on a personal dashboard showing all their submissions. The users could also cancel a running submission if (based on the returned log file) they felt the model was not producing useful results and wished to limit the usage of their time quota (described below).

© 2020 Schaffter T et al. *JAMA Network Open.*

eFigure 4. Participant submission workflow during the DM Challenge.

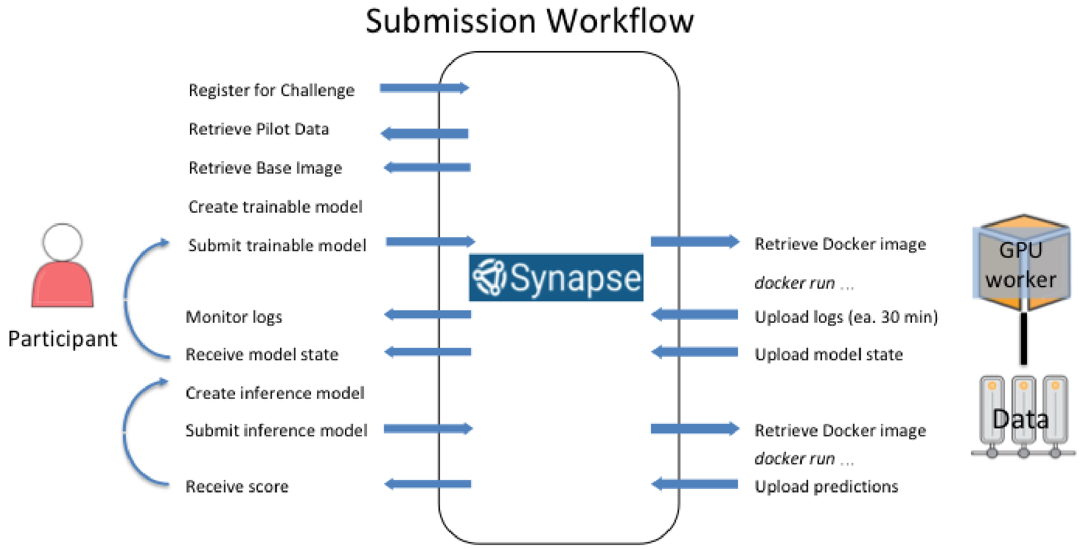

After visiting the Synapse web site to learn about the Challenge and register, participants had access to Challenge data, example models, and GitHub links to source code. After developing a candidate model, participants pushed the Docker image to Synapse, where a batch submission queue retrieved it, mounted the Challenge training data and executed it, returnied progress, logs and finally the trained model state. A similar process for inference submissions led to a score on the Challenge leaderboard. Training and inference were iterative within each round. The Challenge culminated with a final round to determine the best performers.

Once a training submission was completed, the model state (up to 20GB) was uploaded to Synapse and the submitter received an email notification, including the size of the captured model state file, any error information (if the model encountered an error) and a signed token, which could be used to retrieve the model state. Additionally, log files from the running containers were archived to Synapse and shared with the participants. After one or more training submissions, when a participant was satisfied with the performance of the trained model, they prepared an inference submission for evaluation, using (1) a chosen model state generated during training (specified by the ID of the Synapse file to which it was uploaded) and (2) the Docker image for the model itself. The file has the form:

```
model_state=syn12345
inference=docker.synapse.org/syn4224222/dm-train-caffe@sha256:c129...ff44
```

Models sent to this queue were run against a validation set and wrote their predictions to a specified output file, as shown in **eFigure 5**. The predictions were scored, and the scores posted to the Challenge leaderboard. To ensure isolation of validation data, neither log files nor predictions were returned to the participant. Only in the case that the model encounters an error are the last few lines of the log files returned, to assist in debugging. Each participant was limited to three inference submissions (to each of two Sub-challenges) in each five-week round. At the end of the round all scores were published to the leaderboard. In the final round each team had just one chance to submit an inference submission and the results determined the Challenge top performers.

© 2020 Schaffter T et al. *JAMA Network Open.*

eFigure 5. Execution of inference submissions.

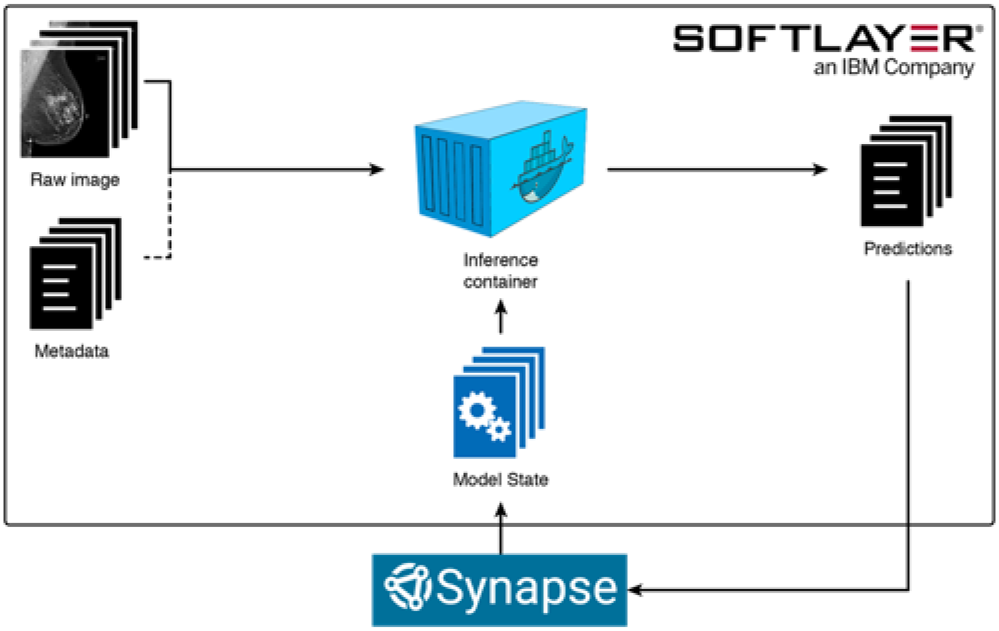

A participant's Docker container was started on a GPU server, mounting the model state created during training and stored to Synapse. Validation data were presented to the model and its predictions captured for scoring against the gold standard. To protect the validation data, user feedback was minimized. The user could view their model's progress and ultimately their score as well as the tail of their log file, should an error occur. No other information was revealed.

Express lanes

A queue was established for submitting models for training and for submitting inference models for each of the two Sub-challenges. At peak times (generally the final days of each round) queues could be backed up for days. This caused a problem for erroneous models: A participant might wait for days only to discover their submitted model had an error that prevented it from running. To address this problem, we created so-called "express lane" versions of each submission queue in which both the data were small (the downloadable pilot data was used) and execution time was limited to thirty minutes. Thus, a participant had some assurance that a long-enqueued submission would run to completion once execution began.

Compute

The compute servers used in the Challenge featured NVIDIA Tesla K80 GPUs. We divided the physical resources of each server to give exclusive hardware to each of two models, running in parallel (with some resources reserved for our supervisory processes). Each submitted model had exclusive access to the following:

- One Tesla K80 GPU Card (2 Kepler GK210 GPUs, each having 2496 cores and 12 GB of memory)
- 22 Intel CPUs
- 200GB memory
- 200GB of scratch space on local storage

© 2020 Schaffter T et al. *JAMA Network Open.*

- 20GB of local storage for the trained *model state*, to be returned to the participant

The machines provisioned for training provided 10TB of local storage per team for preprocessed data, as explained earlier. The machines provisioned for inference required no such local storage.

Data hosting was different in the two clouds, though the difference was invisible to the participants. The IBM Cloud servers had considerable local storage per machine, and the Challenge data was replicated onto each physical server to maximize access of data by the GPUs. On the Amazon cloud we used the Elastic File System (EFS) service, a virtual network share, mounted to the GPU servers, which scaled in bandwidth according to the amount of hosted data. Both approaches achieved high performance transfer of the Challenge image data to the GPU-based models.

Quotas

To fairly share finite Challenge resources, we imposed use limits on participants: Training submissions were limited to two weeks (336 hours) of cumulative wall time, while inference submissions, while unlimited in time, were limited in count to three submissions per team in each round to prevent overfitting. The submissions were serviced by a pool of the previously described GPU-servers. To ensure that teams received their compute share, administrators monitored the backlog and added servers to the worker pool as the submission queue grew. The architecture allowed us to fluidly scale, adding servers as demand grew and retiring servers when not needed.

There was a requirement not to let participants download the Challenge data. All models were run without network access to ensure submitted models did not upload Challenge data to remote systems across the internet. All communication between the model and the outside world was through mounted disk volumes. However, the use of a batch submission system meant that conduits for returning information to participants were required, specifically for returning log files and model state. Though Challenge participants agreed not to retrieve data files, there was the chance that one might attempt to copy restricted Challenge data into one of the retrieved files. A creative attacker could have further encrypted or otherwise disguised data files to avoid detection during inspection of data in transit. The most straightforward approach to avoid data theft was to monitor the size of retrieved data; thus, we limited log file upload to 1GB/day. We also implemented a strategy by which participants could selectively “spend” their data quota: Rather than return model state to participants directly, we kept the data “locked” and sent a cryptographically signed token in the email notification that training was complete. Participants could exchange the token for access to their model state, the size of which was decremented from their per-round quota. This meant that a participant could retrieve a model state well in excess of 1GB several times in a round, provided their total (along with retrieved log files) did not exceed their 35 GB per round quota. In practice this worked very well, with a total data retrieval, across all teams, less than 100GB per round.

Participants were able to view dashboards of their submissions to all queues (training, inference, regular and express lane). The queues for training submission showed the run time and data retrieval quotas used and remaining. All queues featured a cancel button. Participants could monitor their log files and cancel poorly converging training submissions to preserve their time quota. Although log files were not returned for inference submissions, users could cancel submissions not yet executed allowing them to apply their three-submission limit to improvements conceived after original model submission.

© 2020 Schaffter T et al. *JAMA Network Open.*

Model Reuse

GPU processors were a key element of the compute infrastructure. In Unix they appear as *character devices* that need to be mounted to the participant's Docker container, much as are file shares. The Docker mount parameter provisions GPUs for exclusive use by a model. Moreover, NVIDIA *user libraries* installed on the host must be mounted to the model containers. The libraries (which serve as the gateway to the GPUs) precisely match the version of the GPU but have a generic interface. Thus, by keeping these libraries on the server (rather than installing them in model containers) the models could be reused in the future on servers having later versions of NVIDIA GPUs and libraries. To help determine the correct Docker mount parameters, NVIDIA provides the NVIDIA Docker tool, which interrogates the server for its GPU device addresses and user library location, then generates the correct Docker mount parameters. Using this tool allowed the Challenge system to correctly configure reusable models for server execution.

eAppendix 8. Combining model predictions into ensembles

Through various domains, it has been observed that an ensemble of diverse set of predictors perform more robustly than any of the individual predictor[28,29]. In social sciences this collaborative decision-making process is known as WOC (Wisdom of the Crowds)[30]. In the machine learning literature, the process of combining multiple base classifiers is known as ensemble learning. An ensemble of classifiers is a set of base classifiers whose individual predictions are combined to predict labels of unseen examples. Ensemble learning has also been used in various DREAM Challenges with significant performances improvements over the best individual methods[31-33].

It has been observed that base classifier diversity has a significant effect on the performance of the ensemble classifier[34,35]. During DM Challenge, participants were asked to develop algorithms that took as input the data from a screening exam and provided as output a number between 0 and 1 (the confidence level) indicative of the likelihood predicted by the algorithm that the woman would develop breast cancer within a year from the screening test. Participants applied a variety of approaches, such as different network architectures, use of different public/private datasets or using strongly/weakly labeled data to train their models. This diversity in methodologies resulted in important differences between individual predictive algorithms. We hypothesized that an ensemble of predictors would significantly improve upon the performance of the best individual predictor, and even more so if the radiologist assessment (represented with a 1 if the woman was recalled or a 0 otherwise) was combined with the algorithmic predictions. For this purpose, we generated two ensemble classifiers: one using only the community phase eight top performing algorithms (the CEM ensemble) and the other using the same eight top performing algorithms and the radiologist assessment (the CEM+R ensemble). We used a meta-learning method called stacking to generate the ensemble classifiers[36]. The inputs to the ensemble learner were the confidence level of each of the eight algorithms for the CEM ensemble, and those same confidence levels plus the radiologist assessment for the CEM+R ensemble. To avoid overfitting, we used an elastic net regularized logistic regression[37] as the meta-learner and ten-fold cross validation for parameter tuning. We trained the ensemble classifiers on the KPW training data and evaluated their performance on the KPW evaluation dataset as well as the Karolinska evaluation dataset, which served as an independent test set. We used the R package caret for ensemble construction[38].

Running the ensemble model

We provide a method for executing the ensemble model against a novel data set. This is implemented as a workflow, written in the Common Workflow Language (CWL)[39]. The code and instructions for using it are provided through GitHub[40].

eAppendix 9. Participation in the Challenge

The announcement of the Challenge was well received with 1000 pre-registrants. Ultimately 1272 individuals joined the Challenge. Over the seven-month duration of the competitive phase of the Challenge people came and went, but there were about 400 highly active teams. The total number of submitted models (across both Sub-challenges, both training and inference, both express lane and full data set) are detailed below.

registered teams = 124
participants who are not part of a team = 1017

The participation in Sub-challenge 1 was higher than in Sub-challenge 2. Sub-challenge 1 was the entry point to the DM Challenge with data limited to the images of the last mammography exam of subjects for which predictions were requested. The dataset in Sub-challenge 2 was more complex with the addition of clinical and demographic information as well as information from past exams (if available). The second reason was related to the time quota allotted to each team to train models and the massive size of the training set. Because training models for Sub-challenge 1 already required a large amount of time, teams preferred to focus on this sub-challenge rather than spending their time quota on the second Sub-challenge. While past exams did not have cancer in the Challenge dataset and thus could not provide a useful reference, it may be that participants considered that the most important predictive features were included in the images of the exams for which predictions were asked and less from the clinical/demographic information.

Non-'express lane' submissions (evaluation: SC1 and SC2 together):

Number of submissions	Training submissions	Evaluation submissions
Submitted (may have failed)	4554	709
Completed	2013	305

All submissions (express lane/non-express lane, evaluation: SC1 and SC2 together)

Number of submissions	Training submissions	Evaluation submissions
Submitted (may have failed or halted due to time)	5182	2463
Completed within the abbreviated time window	N/A	650

© 2020 Schaffter T et al. *JAMA Network Open.*

eAppendix 10. Best-performing models submitted at the end of the Competitive Phase

The following sections provide insights into the models developed by the best performers in the DM DREAM Challenge. Additional information about each model, including the submitted docker image and source code, is available on the Challenge website (https://www.synapse.org/Digital_Mammography_DREAM_Challenge).

Therapixel model (Best performer in Sub-challenge 1 and 2)

Unlike most of the other teams, Therapixel's team designed a custom deep learning architecture instead of reusing an existing model such as GoogLeNet[41], VGG Net[42] or AlexNet[43]. This decision was motivated by early experiments where the team trained existing models from scratch on mammography images that achieved relatively poor performance. The reason is because digital mammography images are intrinsically different from real-world images that are targeted by most of the available deep learning models. During the DM Challenge, the team identified and addressed the following issues: the need to work with high resolution images, propagate a very weak learning signal, adapt the architecture to detect objects of interest different from traditional, real-world objects (cars, animals, fruits, etc.), and address the huge class imbalance.

For clearness of the following, we note that Therapixel's model was trained at image (view) level, breast cancer probability was inferred as the average cancer probability of different views, and patient cancer probability was inferred as the max cancer probability of the two breasts.

The size of the images of the DM Challenge ranged from 3328x2560 px (8.5 megapixels) to 5928x4728 px (28 megapixels). In Therapixel's approach the images were down-sampled to 1152x832 px (1 megapixel). This significantly reduced the amount of information to process which; however, was still much larger than the size of the input layer of GoogLeNet and VGG Net (224x224 or 0.05 megapixels) or AlexNet (227x227). It was clearly not enough; for example, one of the two types of cancer that teams have been tasked to detect (DCIS cancer) is characterized by very small micro-calcifications located in the milk ducts. Resizing mammography images to match the size of the input layer of available deep learning models would then lead to a loss of information and the inability to detect DCIS cancer. To address this issue, the team deeply modified the VGG Net architecture main modifications being as follows:

- Reducing the number of parameters, since the original architecture had too big memory footprint to train on high resolution images.
- Using 6 pooling layers instead of 5 to better fit the (usually small) lesions.
- Inserting a final (7th) pooling layer with large receptive field before the last three layers (which infer the cancer probability) of the network to be more robust with respect to lesion position.

© 2020 Schaffter T et al. *JAMA Network Open.*

eFigure 6. Architecture of the deep neural network implemented by the team Therapixel at the end of the competitive phase of the Challenge.

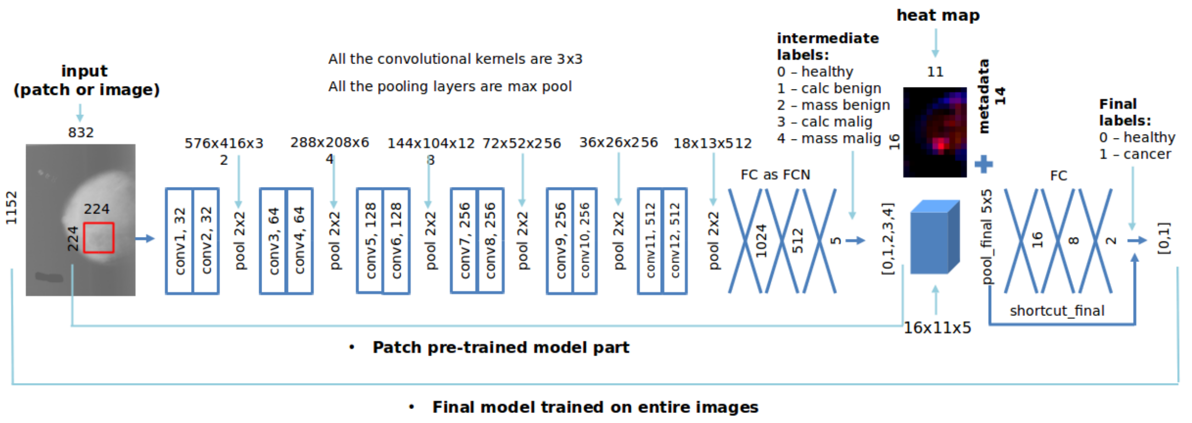

For Sub-challenge 2 the team used patient metadata as an additional input vector concatenated with the feature map deep inside the network (see "metadata" on Fig S6). The previous exams of the same patient were not used due to lack of computational time.

A second key component implemented by the team was to pre-train the first part of the network on strongly-labeled data; that is, where the location of lesions is specified. Because such information was not part of the Challenge data, the team pre-trained their model on patches (region of interest around the lesion) from the DDSM dataset[12]. Then, this network trained on patches was applied to entire images to produce a coarse heat map of different types of lesions (healthy, calcification benign, etc., see "Intermediate labels" on Fig S6). Finally, three more layers were added on top of this heat map and trained with image-wise labels to infer final cancer probability. This 2-step (patch-full image) training procedure enables to propagate a very weak learning signal through a relatively deep model that has 18 processing layers in total.

This DDSM-trained network when applied directly to DREAM images gives performance of about 60-65% in terms of breast-level AUC. We explain this poor performance by very different distributions of pixel intensities between DDSM and DREAM images. The difference in the distribution of pixel intensities originates from the fact that DDSM is a database of digitized mammograms (that is, they were originally film-screen mammograms) and are not intrinsically digital images like the ones DREAM used. Examples of images from the two datasets are given in **eFigure 7**. However, when fine-tuning on the DREAM images, the model quickly adjusts to the new image appearance, training much faster than from scratch and achieving a better generalization.

© 2020 Schaffter T et al. *JAMA Network Open.*

eFigure 7. Comparison between a scanned, film mammogram image from DDSM dataset (left) and a digital mammogram images from the DM Challenge dataset provided by KPW (right).

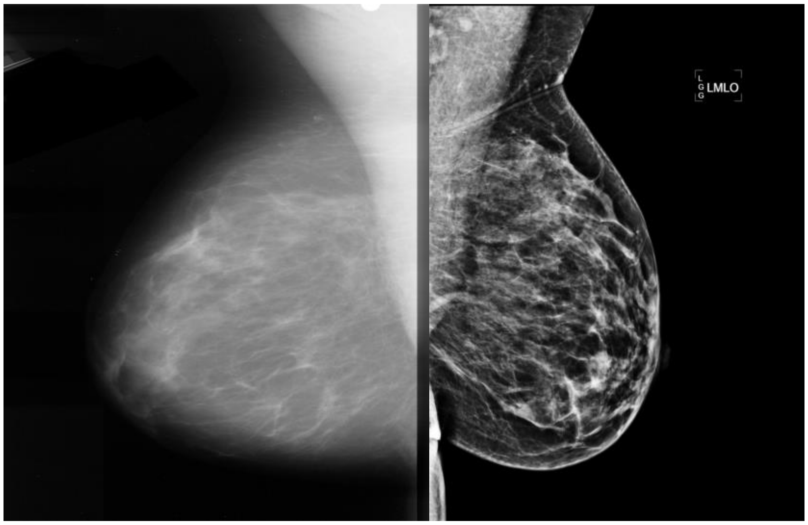

Finally, the huge class imbalance was addressed by training on mini-batches containing the same number of positive and negative examples. Such a simple strategy permits to hide the true distribution and was empirically found to better optimize the AUC metric prioritizing it over the classification accuracy.

The custom network architecture and training procedure described above, with the help of manual fine-tuning steps such as appropriate learning rate, usage of exponential moving averages and model ensembling enabled the model of the Therapixel's team to achieve an AUC score of 0.858 (exam level) and 66.3% specificity at sensitivity 85.9% on KPW Validation set (Sub-challenge 2).

Even though the model suggested by Therapixel's team is a classification network, the model can still be used to extract some information about the location of potential malignant lesions inside the breast. By applying the method described in[44] to a deep learning network, a saliency map of the same size as the input image can be generated for each class. The intensity of a given pixel in a saliency map represents the predicted probability that this pixel is associated to the class of interest.

Dezso Ribli's model (2nd Best performer in Sub-challenge 1)

Mammograms are huge images, while cancers only correspond to a relatively small area of the image (1-2%); therefore, the team reformulated the task as object detection instead of the original image classification task of the Challenge. A high performing object detector model based on deep convolutional neural networks, Faster-RCNN[45], was adapted to the problem and trained on mammogram images with cancer location annotation (bounding boxes, see eFigure 8). The main Challenge dataset had no location annotation; therefore, Faster-RCNN could only be trained on publicly available mammograms from DDSM[12], INbreast[13] and a very small set of DREAM pilot images which was annotated by hand. The main Challenge dataset was only used for validation and model selection. In the collaborative phase of the Challenge new datasets became available, and the models were trained on the DDSM and the Optimam dataset and a small dataset from a Hungarian university[46].

© 2020 Schaffter T et al. *JAMA Network Open.*

eFigure 8. Outline of the Faster-RCNN approach for mammography. Reprinted with permission from[46].

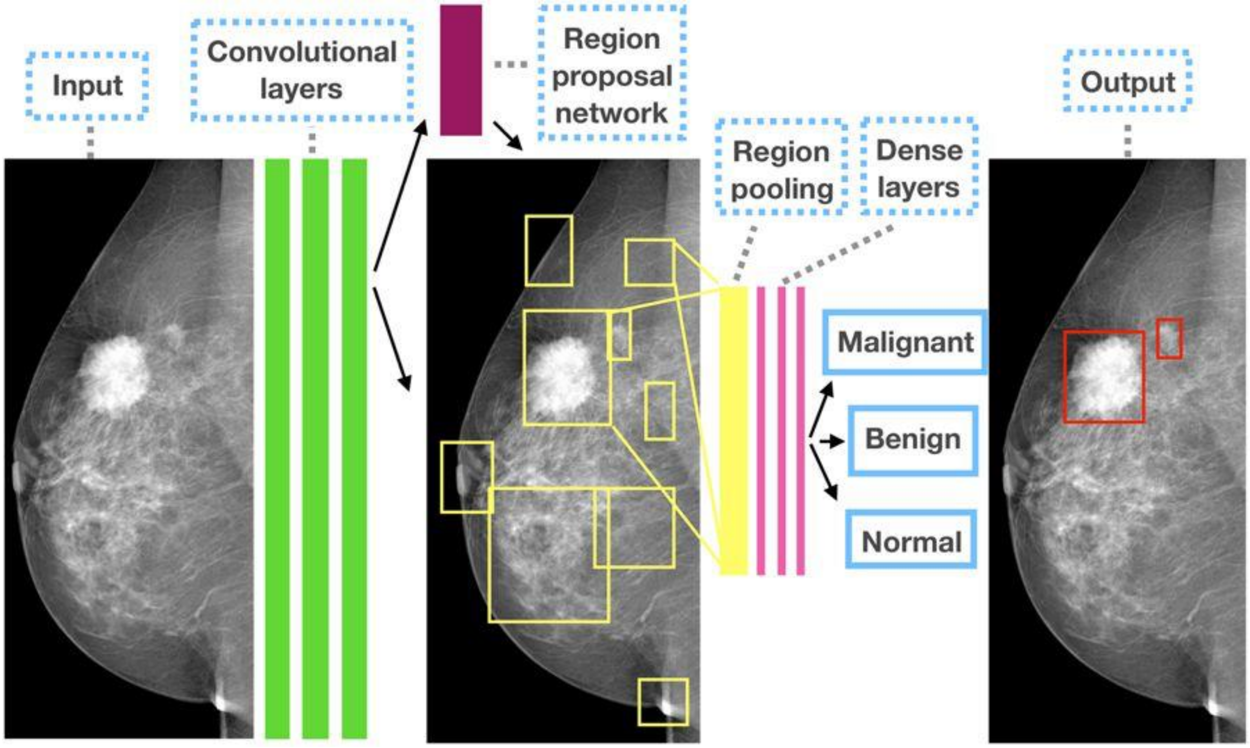

The final score assigned to an image was the score of the most confident localized cancer detection. The score of a breast was an average score of all views, a score on an exam was the maximum of the scores of the laterality. When ensembling models, the scores of different models were averaged on the image level. Further details about this approach can be found in a separate paper[46].

At the end of the Challenge this solution was found to be highest performing approach. In the collaborative phase of the Challenge the method reached the highest AUC at breast level both on the Challenge dataset, and the Karolinska dataset, AUC = 0.893 and 0.928 respectively. In the competitive phase of the Challenge, the method reached the 2nd position in Sub-challenge 1 with AUC = 0.85.

An important aspect of this approach is that Faster-RCNN is based on the precise localization of cancerous lesions, which enables the method to be directly used as a computer aided detection tool unlike any other best performing methods which are based on classifying whole images.

Yuanfang Guan's model (3rd Best performer in Sub-challenge 1)

The color profiles between the DM data and the training data used by this team (INBreast and BCDR) were different; thus, this team first mapped the color profiles through sigmoid transformation and percentile fitting. Then they trained two types of models by segmentation networks. The first model predicted calcification and the number of calcifications in a small patch. This team trained full resolution model on calcification counts in a small region in the first model that allowed detection of DCIS. Then they trained a low-resolution mass model and used mass size as feature.

DeepHealth's model (4th Best performer in Sub-challenge 1)

To address the dependency of cancer/no-cancer status on highly localized regions, DeepHealth's developed a two-stage training approach, consisting of first training patch-level classifiers, which were then used to initialize a fully-convolutional image-level classifier. For the patch-level training, the DDSM dataset was used to construct a training set of cropped image patches, using the available lesion segmentation masks and pathology results to generate labels. Variants of ResNet CNNs[47], particularly the Wide ResNet formulation[48] with custom hyperparameters, were used as the patch classifiers. Patch CNNs were trained at two scales to account for the wide range of scales of lesions-varying from small microcalcifications needing fine detail for diagnosis, to larger masses requiring bigger spatial extents. Specifically, 256x256 cropped patches were created from images originally resized to ~2750x1500 and ~1100x600. Random size augmentation of 15% was used in the initial resizing of the image, i.e. images were randomly resized to 85-115% of the specified target size before patch cropping. Random rotations up to 30 degrees and horizontal mirroring were also used for data augmentation. The CNN trained on the larger image size was trained for calcification classification, whereas the CNN trained on the smaller image size was trained for mass classification. After patch-level training, the CNNs were used in a scanning-window (i.e. convolutional) fashion to initialize a full-image model that could then be trained in an end-to-end fashion using binary image-level labels. The full-image model consisted of global average pooling on the convolutional feature maps produced by each patch CNN, followed by concatenation of the two resulting feature vectors and a single, fully-connected classification layer. This model was again trained on the DDSM dataset, and then fine-tuned on the DREAM images. A schematic of the training procedure is shown in **eFigure 9**.

© 2020 Schaffter T et al. *JAMA Network Open.*

eFigure 9. For the DREAM Challenge, predictions were made on a single-image basis and averaged across views to generate breast-level scores.

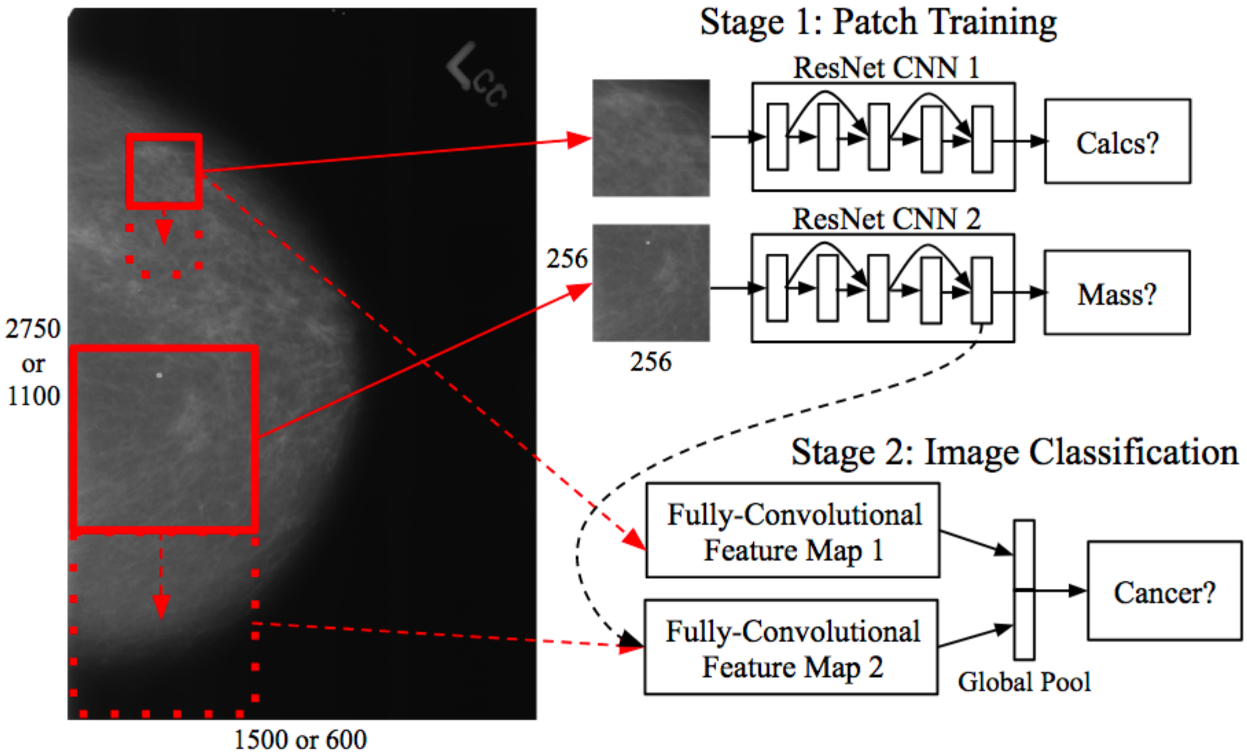

In the competitive phase, the DeepHealth model scored 0.843 AUC. In the post-competitive phase, the model reached 0.902 AUC following several modifications, including image background trimming, 1:1 class balancing, a single input scale with height of 1750, and pre-training on the Optimam dataset.

The DeepHealth image-level model was trained one image at a time (batch size of 1), with the positive images oversampled from their natural proportion at a rate of 1 positive image chosen for every 3 cancer-negative images. To combat overfitting, data augmentation of random horizontal mirroring and resizing were again used at the image level. Predictions were made on a single-image basis and averaged across views to generate breast-level scores. In SC2, an adaboost classifier was independently trained on the exam metadata and combined with the image model via a weighted average of the model outputs.

Strategies associated with high performance

Among the 26 teams who responded to our survey, 14 teams (54%) reported using public or private datasets to pre-train their model on strongly labeled samples; that is, mammogram images that have annotation as to the precise location of abnormal lesions within the image. We observed that algorithms that included training on strongly labelled data reached a significantly higher performance in the KPW evaluation data than methods that use only weakly labelled data (P=0.017, see **eFigure 10A**). Additionally, these high performing teams used an ensemble learning strategy, representing 46% of all methods, achieved a significantly higher performance (P=0.012, see **eFigure 10B**). This is consistent with results from other DREAM Challenges: teams that use ensemble learning tend to reduce overall variance in performance metrics and stable and generalizable results [31].

© 2020 Schaffter T et al. *JAMA Network Open.*

eFigure 10. Area under the curve (AUC) of the methods that have been reported as A) having been trained on strongly labelled data (private or public datasets) and B) using an ensemble of models instead of a single model in the Validation Phase of the Challenge. Both approaches highlight a significant improvement in AUC (P<0.05).

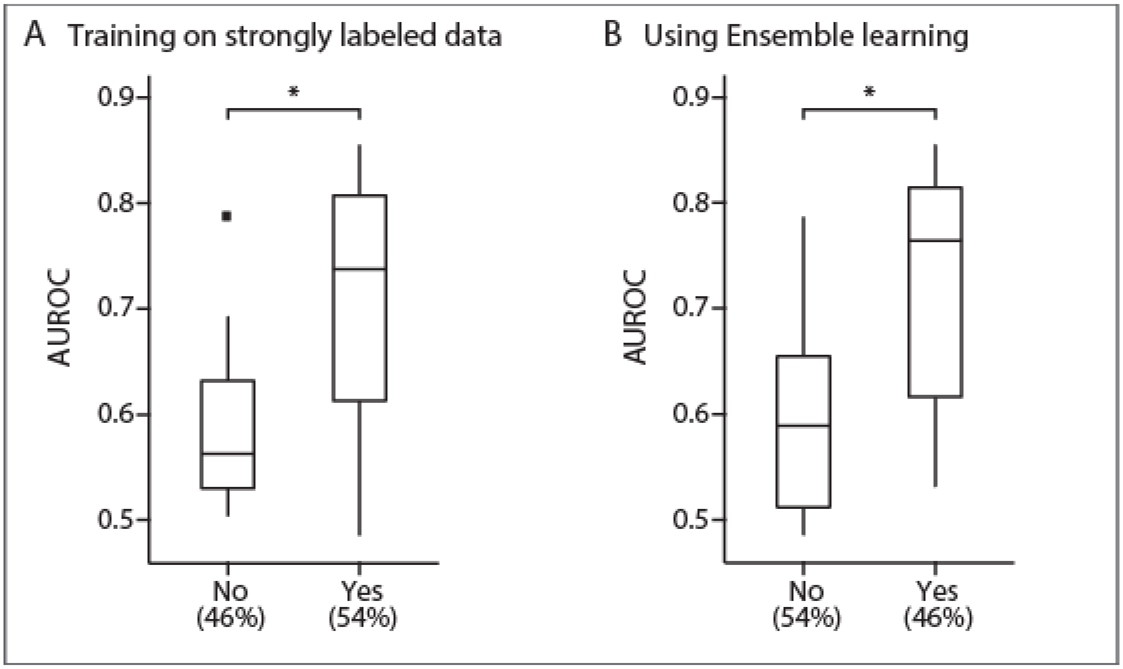

© 2020 Schaffter T et al. *JAMA Network Open.*

eAppendix 11. DM DREAM Consortium

The following participants are members of the DM DREAM Consortium:

Christoph M. Friedrich, PhD. University of Applied Sciences and Arts Dortmund, Department of Computer Science, Emil-Figge-Str. 42, 44227 Dortmund, Germany

Lester Mackey, Ph.D. Microsoft Research New England, 1 Memorial Drive, Cambridge, MA 02142.

Hossein Azizpour, PhD. KTH, Division of Robotics, Perception, and Learning, Stockholm, Sweden.

Joyce Cahoon, M.S. North Carolina State University, 2311 Stinson Dr, Raleigh, NC 27695 USA.

Kevin Smith, Ph.D. 1) School of Electrical Engineering and Computer Science, KTH Royal Institute of Technology, Stockholm, Sweden, 2) Science for Life Laboratory, Solna, Sweden.

Bibo Shi, Ph.D. Carl E. Ravin Advanced Imaging Laboratories, Department of Radiology, Duke University School of Medicine, Durham, NC 27705 USA.

Li Shen, Ph.D. Icahn School of Medicine at Mount Sinai, 1425 Madison Ave, New York, NY 10029 USA.

Jae Ho Sohn, MD, MS. University of California San Francisco, Radiology and Biomedical Imaging, 505 Parnassus Ave, San Francisco, CA 94143 USA.

Hari Trivedi, M.D. Emory University, 1364 Clifton Road Northeast, Atlanta, GA 30322 USA.

Yiqiu Shen. New York University, 60 5th Ave, New York, NY 10011, USA.

Ljubomir Buturovic, Ph.D. Clinical Persona Inc., 932 Mouton Circle, East Palo Alto, CA 94303, USA.

Jose Costa Pereira, Ph.D. INESCTEC, Rua Dr. Roberto Frias, 4200-465 Porto, Portugal.

Jaime S. Cardoso, Ph.D. INESC TEC and University of Porto, Porto, Portugal.

Michael Kawczynski, M.S. Bakar Computational Health Sciences Institute, University of California, San Francisco, 550 16th Street, San Francisco, CA 94158 USA.

Eduardo Castro, MSc. INESC TEC, Rua Dr. Roberto Frias, Campus da Faculdade de Engenharia da Universidade do Porto, 4200-465 Porto Portugal.

Karl Trygve Kalleberg, MD, PhD. KolibriFX AS, Gaustadalléen 23A, 0373 Oslo, Norway.

Obioma Pelka, M.Sc. 1) Department of Computer Science, University of Applied Sciences and Arts Dortmund, Germany, 2) Department of Diagnostic and Interventional Radiology and Neuroradiology, University Hospital Essen, Germany.

Imane Nedjar, M.Sc. Biomedical Engineering Laboratory Tlemcen University, 22 rue Abi Ayed Abdelkrim, Tlemcen 13000, Algeria.

Kyunghyun Cho, Ph.D. New York University, 60 5th Ave., New York, NY 10012, USA.

Krzysztof J. Geras, Ph.D. Department of Radiology, NYU School of Medicine, 660 1st Avenue, New York, NY 10016, USA.

Felix Nensa, M.D. Department of Diagnostic and Interventional Radiology and Neuroradiology, University Hospital Essen, Hufelandstr. 55, 45147 Essen, Germany.

B.E. Ethan Goan, Ph.D. Queensland University of Technology, 2 George St, Brisbane, QLD, 4000 Australia.

Sven Koitka, M.Sc. 1) Department of Computer Science, University of Applied Sciences and Arts Dortmund, Dortmund, Germany, 2) Department of Diagnostic and Interventional Radiology and Neuroradiology, University Hospital Essen, Essen, Germany.

Can Son Khoo, BSc. University College London, United Kingdom.

Luis Caballero, Ph.D. Instituto de Fisica Corpuscular, C/ Catedratico Jose Beltran 2, 46980, Paterna, Valencia, Spain.

Joseph Y. Lo, Ph.D. Department of Radiology, Duke University School of Medicine, Durham, NC 27705, USA.

David D. Cox, Ph.D. MIT-IBM Watson AI Lab, IBM Research, Cambridge, MA 02142 USA.

Pavitra Krishnaswamy, Ph.D. Institute for Infocomm Research, A*STAR, 1 Fusionopolis Way, #21-01 Connexis (South Tower), Singapore 138632.

A. Gregory Sorensen MD. DeepHealth, Inc., 1000 Massachusetts Avenue, Cambridge MA 02138.

Hwejin Jung, Ph.D. Korea University, Seoul, Republic of Korea.

Bibo Shi, Ph.D. Carl E. Ravin Advanced Imaging Laboratories, Department of Radiology, Duke University School of Medicine, Durham, NC 27705 USA.

© 2020 Schaffter T et al. *JAMA Network Open.*

Gerard Cardoso Negrie, M.Sc. Satalia, 40 Islington High Street, N1 8EQ London, UK.
Michael Kawczynski, M.S. Bakar Computational Health Sciences Institute, University of California, San Francisco, 550 16th Street, San Francisco, CA 94158 USA.
Kyunghyun Cho, Ph.D. New York University, 60 5th Ave., New York, NY 10012, USA.
Can Son Khoo BSc. University College London, United Kingdom. can.khoo.10@ucl.ac.uk
Joseph Y. Lo, Ph.D. Department of Radiology, Duke University School of Medicine, Durham, NC 27705, USA.

© 2020 Schaffter T et al. *JAMA Network Open.*

eReferences

1. Kaufman S, Rosset S, Perlich C, Stitelman O. Leakage in Data Mining: Formulation, Detection, and Avoidance. *ACM Trans Knowl Discov Data*. 2012;6(4):15:1–15:21.
2. Keller BM, Nathan DL, Wang Y, et al. Estimation of breast percent density in raw and processed full field digital mammography images via adaptive fuzzy c-means clustering and support vector machine segmentation. *Med Phys*. 2012;39(8):4903–17.
3. Nisbet R, Elder J, Miner G. Handbook of Statistical Analysis and Data Mining Applications. Academic Press; 2009.
4. McCormack VA, dos Santos Silva I. Breast density and parenchymal patterns as markers of breast cancer risk: a meta-analysis. *Cancer Epidemiol Biomarkers Prev*. 2006;15(6):1159–69.
5. Levy PS, Lemeshow S. Sampling of Populations: Methods and Applications. John Wiley & Sons; 2013.
6. Kaiser Foundation Health Plan of Washington. Breast cancer screening https://wa.kaiserpermanente.org/healthAndWellness/index.jhtml?item=%2Fcommon%2FhealthAndWellness%2Ftests%2Fscreenings%2Fmammogram.html. Accessed August 30, 2019.
7. American College of Radiology. ACR BI-RADS Atlas® 5th Edition. https://www.acr.org/Clinical-Resources/Reporting-and-Data-Systems/Bi-Rads. Accessed August 30, 2019.
8. Lehman CD, Arao RF, Sprague BL, Lee JM, Buist DS, Kerlikowske K, et al. National Performance Benchmarks for Modern Screening Digital Mammography: Update from the Breast Cancer Surveillance Consortium. *Radiology.* 2017;283(1):49-58.
9. Lind H, Svane G, Kemetli L, Törnberg S. Breast Cancer Screening Program in Stockholm County, Sweden - Aspects of Organization and Quality Assurance. Breast Care. 2010;5(5):353–7.
10. Törnberg S, Kemetli L, Ascunce N, Hofvind S, Anttila A, Sèradour B, et al. A pooled analysis of interval cancer rates in six European countries. Eur J Cancer Prev. 2010;19(2):87–93.
11. Houssami N, Hunter K. The epidemiology, radiology and biological characteristics of interval breast cancers in population mammography screening. NPJ Breast Cancer. 2017;3:12.
12. Heath M, Bowyer K, Kopans D, Moore R, Kegelmeyer WP. The digital database for screening mammography. In: Proceedings of the 5th international workshop on digital mammography. Medical

© 2020 Schaffter T et al. *JAMA Network Open.*

Physics Publishing; 2000. p. 212–8.

13. Moreira IC, Amaral I, Domingues I, Cardoso A, Cardoso MJ, Cardoso JS. INbreast: toward a full-field digital mammographic database. Acad Radiol 2012;19(2):236–48.
14. Suckling J, Parker J, Dance D, et al. The mammographic image analysis society digital mammogram database. In: Exerpta Medica. International Congress Series. 1994. p. 375–8.
15. Halling-Brown MD, Looney PT, Patel MN, Warren LM, Mackenzie A, Young KC. Mammographic Image Database (MIDB) and Associated Web-Enabled Software for Research. In: Breast Imaging. Springer International Publishing; 2014. p. 514–9.
16. Petrick N, Sahiner B, Armato SG, Bert A, Correale L, Delsanto S, et al. Evaluation of computer-aided detection and diagnosis systems. Med Phys. 2013;40(8).
17. Gallas BD, Chan H-P, D’Orsi CJ, Dodd LE, Giger ML, Gur D, et al. Evaluating imaging and computer-aided detection and diagnosis devices at the FDA. Acad Radiol. 2012;19(4):463–77.
18. Kass RE, Raftery AE. Bayes Factors. J Am Stat Assoc. 1995;90(430):773–95.
19. Lehman CD, Arao RF, Sprague BL, Lee JM, Buist DS, Kerlikowske K, et al. National Performance Benchmarks for Modern Screening Digital Mammography: Update from the Breast Cancer Surveillance Consortium. Radiology. 2017;283(1):49–58.
20. SAGE Bionetworks. Synapse. http://synapse.org. Accessed August 30, 2019.
21. Docker Inc. Docker. https://www.docker.com/. Accessed August 30, 2019.
22. Docker Inc. Docker Hub. https://hub.docker.com/. Accessed August 30, 2019.
23. Red Hat. Red Hat Quay.io. https://quay.io/. Accessed August 30, 2019.
24. Yangqing Jia. Caffe. http://caffe.berkeleyvision.org/. Accessed August 30, 2019.
25. TensorFlow. TensorFlow. https://www.tensorflow.org/. Accessed August 30, 2019.
26. LISA lab. Theano. http://www.deeplearning.net/software/theano/. Accessed August 30, 2019.
27. The Apache Software Foundation. Apache MXNet (Incubating). http://mxnet.incubator.apache.org/. Accessed August 30, 2019.
28. Whalen S, Pandey OP, Pandey G. Predicting protein function and other biomedical characteristics with heterogeneous ensembles. Methods. 2016;93:92–102.
29. Martire KA, Growns B, Navarro DJ. What do the experts know? Calibration, precision, and the wisdom

© 2020 Schaffter T et al. *JAMA Network Open.*

of crowds among forensic handwriting experts. Psychon Bull Rev. 2018;25(6):2346-2355.

30. Surowiecki J. The Wisdom of Crowds. Anchor; 2005.

31. Saez-Rodriguez J, Costello JC, Friend SH, Kellen MR, Mangravite L, Meyer P, et al. Crowdsourcing biomedical research: leveraging communities as innovation engines. Nat Rev Genet. 2016;17(8):470–86.

32. Marbach D, Costello JC, Küffner R, Vega NM, Prill RJ, Camacho DM, et al. Wisdom of crowds for robust gene network inference. Nat Methods. 2012;9(8):796–804.

33. Choobdar S, Ahsen ME, Crawford J, et al. Open Community Challenge Reveals Molecular Network Modules with Key Roles in Diseases. bioRxiv 265553. 2018.

34. Banfield RE, Hall LO, Bowyer KW, Philip Kegelmeyer W. Ensemble diversity measures and their application to thinning. Inf Fusion. 2005;6(1):49–62.

35. Ahsen ME, Vogel R, Stolovitzky G. Unsupervised Evaluation and Weighted Aggregation of Ranked Predictions. arXiv preprint arXiv:180204684 2018.

36. Wolpert DH. Stacked generalization. Neural Netw. 1992;5(2):241–59.

37. Friedman J, Hastie T, Tibshirani R. Regularization Paths for Generalized Linear Models via Coordinate Descent. J Stat Softw. 2010;33(1):1–22.

38. Kuhn M. A Short Introduction to the caret Package. R Found Stat Comput. 2015;1–10.

39. CWL Leadership Team. Common Workflow Language. https://www.commonwl.org/. Accessed August 30, 2019.

40. Sage Bionetworks. Digital Mammography DREAM Challenge. https://sagebionetworks.org/research-projects/digital-mammography-dream-challenge/. Accessed August 30, 2019.

41. Szegedy C, Liu W, Jia Y, et al. Going deeper with convolutions. In: Proceedings of the IEEE conference on computer vision and pattern recognition. 2015. p. 1–9.

42. Simonyan K, Zisserman A. Very Deep Convolutional Networks for Large-Scale Image Recognition [Internet]. arXiv [cs.CV]. 2014;Available from: http://arxiv.org/abs/1409.1556

43. Krizhevsky A, Sutskever I, Hinton GE. ImageNet Classification with Deep Convolutional Neural Networks. In: Pereira F, Burges CJC, Bottou L, Weinberger KQ, editors. Advances in Neural Information Processing Systems 25. Curran Associates, Inc.; 2012. p. 1097–105.

© 2020 Schaffter T et al. *JAMA Network Open.*

44. Simonyan K, Vedaldi A, Zisserman A. Deep Inside Convolutional Networks: Visualising Image Classification Models and Saliency Maps. arXiv [cs.CV]. 2013.

45. Cornell University. Faster R-CNN: Towards Real-Time Object Detection with Region Proposal Networks. https://arxiv.org/abs/1506.01497. Accessed August 30, 2019.

46. Ribli D, Horváth A, Unger Z, Pollner P, Csabai I. Detecting and classifying lesions in mammograms with Deep Learning. Sci Rep. 2018;8(1):4165.

47. Cornell University. Deep Residual Learning for Image Recognition. https://arxiv.org/abs/1512.03385. Accessed August 30, 2019.

48. Cornell University.Wide Residual Networks. https://arxiv.org/abs/1605.07146. Accessed August 30, 2019.

© 2020 Schaffter T et al. *JAMA Network Open.*
